# Catalysis of non-canonical protein ubiquitylation by the ARIH1 ubiquitin ligase

**DOI:** 10.1042/BCJ20230373

**Published:** 2023-11-17

**Authors:** Nicholas Purser, Ishita Tripathi-Giesgen, Jerry Li, Daniel C. Scott, Daniel Horn-Ghetko, Kheewoong Baek, Brenda A. Schulman, Arno F. Alpi, Gary Kleiger

**Affiliations:** 1 Department of Chemistry and Biochemistry, University of Nevada, Las Vegas, Las Vegas, NV, U.S.A.; 2 Department of Molecular Machines and Signaling, Max Planck Institute of Biochemistry, Martinsried, Germany; 3 Department of Structural Biology, St. Jude Children's Research Hospital, Memphis, TN 38105, U.S.A.

**Keywords:** proteolysis, ubiquitin ligases, ubiquitins

## Abstract

Protein ubiquitylation typically involves isopeptide bond formation between the C-terminus of ubiquitin to the side-chain amino group on Lys residues. However, several ubiquitin ligases (E3s) have recently been identified that ubiquitylate proteins on non-Lys residues. For instance, HOIL-1 belongs to the RING-in-between RING (RBR) class of E3s and has an established role in Ser ubiquitylation. Given the homology between HOIL-1 and ARIH1, an RBR E3 that functions with the large superfamily of cullin-RING E3 ligases (CRLs), a biochemical investigation was undertaken, showing ARIH1 catalyzes Ser ubiquitylation to CRL-bound substrates. However, the efficiency of ubiquitylation was exquisitely dependent on the location and chemical environment of the Ser residue within the primary structure of the substrate. Comprehensive mutagenesis of the ARIH1 Rcat domain identified residues whose mutation severely impacted both oxyester and isopeptide bond formation at the preferred site for Ser ubiquitylation while only modestly affecting Lys ubiquitylation at the physiological site. The results reveal dual isopeptide and oxyester protein ubiquitylation activities of ARIH1 and set the stage for physiological investigations into this function of emerging importance.

## Introduction

The ubiquitin-proteasome system is a fascinating pathway that controls the biological fates of thousands of eukaryotic proteins [1,2]. Ubiquitylation describes the transfer of ubiquitin, a highly conserved, 76 amino acid protein, to a protein substrate involving a common cascade of enzymes termed E1, E2 and E3 [3]. Ubiquitin must first be activated through the ATP-dependent action of the ubiquitin-activating enzyme (E1). The E1 ∼ ubiquitin (where ∼ represents a high-energy thioester bond between the ubiquitin C-terminus and a Cysteine (Cys) residue in the E1 active site) then transfers the ubiquitin to a ubiquitin-conjugating enzyme (E2) while sustaining the thioester bond [4]. In most cases, the E2 ∼ ubiquitin is then recognized by a ubiquitin ligase (E3) that binds to the protein substrate while also activating the E2 ∼ ubiquitin to promote the transfer of ubiquitin to a Lysine (Lys) residue on the substrate [5].

Although a protein modified with a single ubiquitin can impact the biological fates of proteins such as their cellular localization, it is often the case that the substrate-bound ubiquitin becomes modified by additional ubiquitin protomers resulting in the formation of a poly-ubiquitin chain [6]. While poly-ubiquitylation can signal diverse biological outcomes, protein degradation by the 26S proteasome is the most likely [7]. Poly-ubiquitin chain formation occurs between Lys residues on substrate-bound ubiquitin (often referred to as the acceptor) and an activated donor ubiquitin bound either to the E2 or in some cases E3s (described in detail below). Ubiquitin has seven invariably conserved Lys residues, all shown to participate in poly-ubiquitin chain formation resulting in homotypical chain linkages of distinct structures [8]. Even the N-terminus of ubiquitin may serve as an acceptor of donor ubiquitins to form a poly-ubiquitin chain [9]. Additional complexity has been observed by the mixing of chain-types within a single poly-ubiquitin chain [10], and poly-ubiquitin chain branching occurs when multiple Lys residues on the ubiquitin of a growing chain are ubiquitylated in combination [11]. This dizzying variety of poly-ubiquitin chain topologies affords an array of potential biological outcomes for the ubiquitylated protein that is still very much under scrutiny.

Further adding to the apparent complexity of ubiquitin signaling was the discovery some 18 years ago that residues other than Lys can act as acceptors of ubiquitin [12]. It is now appreciated that Cys, Ser and Thr residues can all be ubiquitylated [13–15]. In the case of Ser and Thr residues, the hydroxy side-chain groups emanating from both protein substrates as well as acceptor ubiquitins [16] may become esterified to the ubiquitin C-terminus. Hydroxy groups on non-proteinaceous, biological molecules such as sugars [17] and lipids [18] can also serve as acceptors of ubiquitin. Not surprisingly, a growing number of E3s have been identified that promote non-canonical ubiquitylation [19–23].

One E3 of particular interest is HOIL-1 which belongs to the RING-in-between RING (RBR) class of ligases [24–26]. Distinct from the more common E1–E2–E3 mechanism described above, RBR ligases first interact with E2 ∼ ubiquitin [27] and catalyze the transfer of ubiquitin to the E3 (rather than directly to the protein substrate). The E3 [28] then catalyzes ubiquitin transfer to either an E3-bound substrate or an acceptor ubiquitin on the substrate to form a poly-ubiquitin chain [29–32]. Several investigations have established that HOIL-1 promotes oxyester bond formation between Ser and Thr residues on both protein substrates as well as on acceptor ubiquitins [17,33]. These results led us to wonder whether HOIL-1 is unique in its ability to catalyze oxyester bond formation or if this may be a more general phenomenon of RBR E3s.

ARIH1 is one of the best-characterized RBRs to date, functioning in Lys-based ubiquitylation with the largest class of E3s in humans, the cullin-RING ligases (CRLs) [34]. There are approximately 300 CRL complexes in humans built around a core scaffold called the cullin. In a majority of CRLs, the cullin forms interactions with the RING-domain containing subunit RBX1 that in combination bind to ARIH1 ∼ ubiquitin. The cullin is also the site of covalent modification by the ubiquitin-like protein NEDD8 [35] (termed neddylation) which both serves to activate the CRL complex as well as to relieve ARIH1 auto-inhibition. Cullins also interact with various protein subunits that are responsible for binding to the protein substrate. These CRL-substrate receptors recognize both the primary structure, and often post-translational modifications in protein substrates that are collectively referred to as degrons [36].

ARIH1 participates in the joining of a donor ubiquitin to an unmodified CRL-bound substrate, an activity often referred to as priming [37–39]. First, auto-inhibited ARIH1 binds to a neddylated CRL which enables the transfer of an activated ubiquitin from the UBE2L3 E2 enzyme to the ARIH1 Rcat domain [30,32,40]. Subsequently, ARIH1 catalyzes the transfer of ubiquitin to CRL-bound substrate. Previous kinetic studies have shown that the transfer rates for ARIH1-catalyzed substrate priming and poly-ubiquitin chain formation are similar enough to form modest poly-ubiquitin chains onto CRL-bound substrates prior to product dissociation from the CRL [32,41]. However, the human E2s UBE2R1 and UBE2R2 catalyze chain elongation on CRL substrates far more rapidly [41], such that ARIH1 and UBE2R-family E2s are thought to collaborate during ubiquitylation of CRL-dependent substrates [29,42]. In summary, ARIH1 is an RBR E3 of great interest due to its clear functional role with CRLs and an intriguing candidate for an E3 that may ubiquitylate protein substrate residues beyond Lys.

## Results

### Ubiquitylation of Ser residues by ARIH1

We began our investigation by monitoring auto-ubiquitylation, the transfer of ubiquitins from an RBR E3 to itself. Recombinant HOIL-1 and an ARIH1 mutant that relieves auto-inhibition (ARIH1 FEE) were first incubated in the presence of E1 enzyme, UBE2L3 and fluorescently labeled ubiquitin, followed by quenching. The samples were then split with half being exposed to hydroxylamine (NH_2_OH) which preferentially hydrolyzes oxyester bonds over Lys-ubiquitin isopeptide bonds.

**Figure 1.**
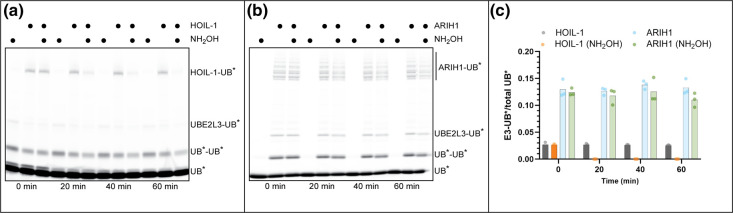
Hydroxylamine sensitivity of HOIL-1 and ARIH1 auto-ubiquitylation indicates the formation of E3-ubiquitin oxyester bonds. (**a**) Fluorescence-scanned SDS–PAGE of auto-ubiquitylation of HOIL-1 and the effect of treatment with hydroxylamine (NH_2_OH). Notice that the signal for HOIL-1-ubiquitin conjugates has nearly disappeared after a 20-min incubation with NH_2_OH. UB* is fluorescently labeled ubiquitin. The time of incubation in the absence or presence of NH_2_OH post-reaction quenching is shown at the bottom of the gel. **(b**) Same as in (**a**) but with an ARIH1 mutant (ARIH1 FEE) that relieves auto-inhibition. (**c**) Graphical representation of the results shown in (**a**) and (**b**). E3-UB*/total UB* represents the amount of labeled ubiquitin attached to E3 compared with total UB* (which includes E3-UB* and UBE2L3-UB* conjugates as well as unanchored mono- (UB*) or di-ubiquitin (UB*-UB*)). The gels are representative of *n* = 3 technical replicates.

HOIL-1 auto-ubiquitylation was apparent (Figure 1a,c), and treatment with NH_2_OH resulted in the complete disappearance of HOIL-1-ubiquitin bands. ARIH1 auto-ubiquitylation was also evident (Figure 1b,c) and with a modest but reproducible reduction in ARIH1-ubiquitin signal also observed relative to the untreated samples. Since much of the ARIH1-ubiquitin that remained after NH_2_OH treatment may be explained by ARIH1 Lys auto-ubiquitylation, a fully reconstituted CRL system was employed since the composition of peptide substrates is easily manipulated.

 Peptide substrates of neddylated CUL1-RBX1 with the FBXW7-SKP1 substrate receptor (hereafter CRL1^FBXW7^) and based on the human CYCLIN E protein (aka CCNE1) were designed to test whether ARIH1 is capable of transferring ubiquitins to Ser residues. A previously reported cyclin E peptide [43] was chosen as an initial template since the sequence encompasses three Ser residues native to CYCLIN E (Ser 381, 387 and 391). The ‘wild-type’ peptide (WT cycE hereafter) is based on residues 377–399 from the human CYCLIN E protein and contained a single N-terminal Lys residue. The N-terminus was acetylated to block the amino group that could in principle also act as a ubiquitin acceptor. To test for Ser ubiquitylation, the Lys residue was replaced with an Arg (KR cycE). To assay for ubiquitylation of only the Lys residue, WT cycE was modified where the three native Ser residues were mutated to Ala or Gly residues (Lys only cycE). Lastly, to ascertain the efficiency of ubiquitylation of a Ser residue located at the native Lys position, the N-terminal Lys residue was replaced with a Ser while all native Ser residues were mutated (Ser only cycE).

**Figure 2.**
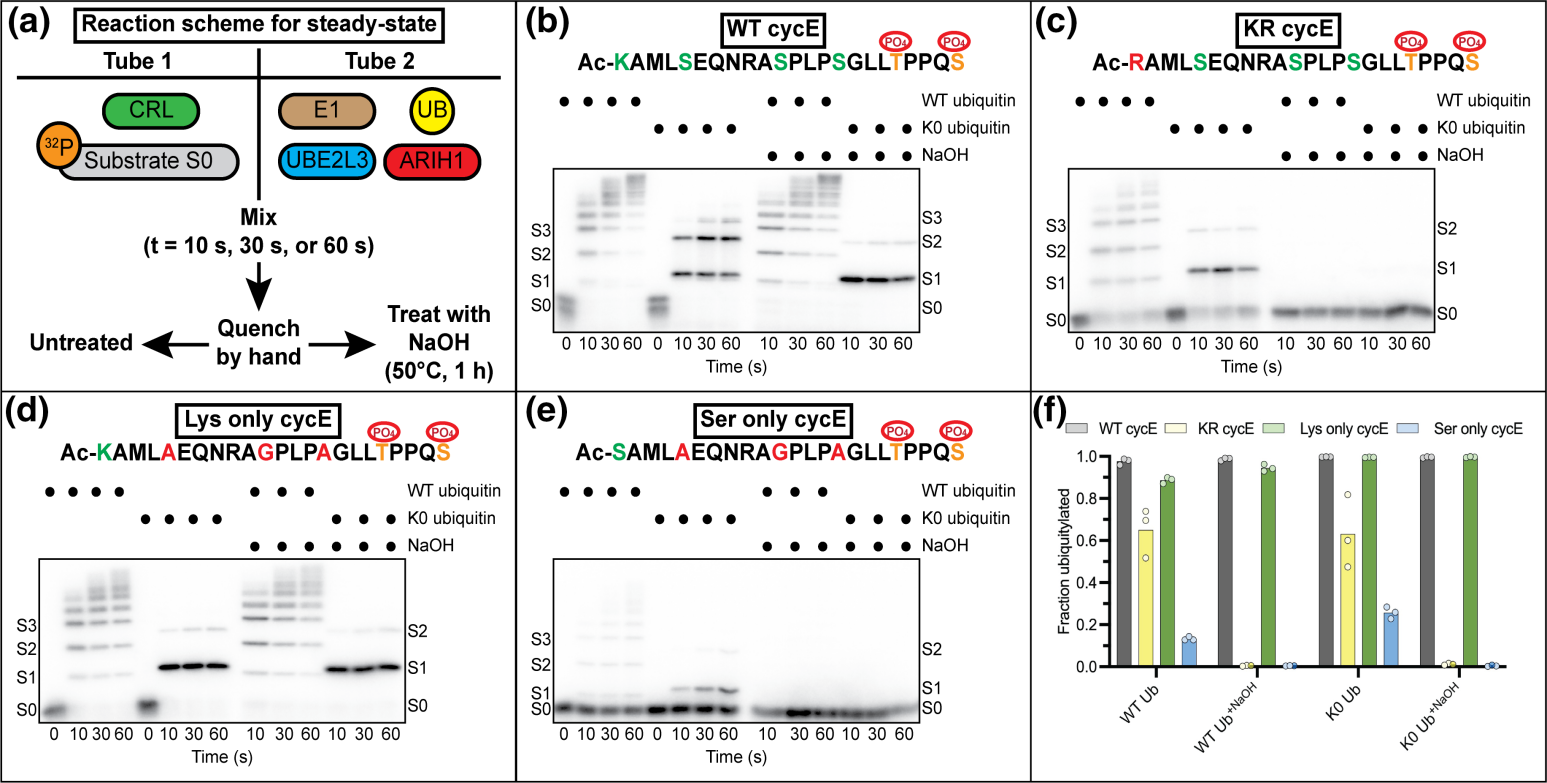
ARIH1 catalyzes the formation of oxyester bonds between ubiquitin and CRL1^FBXW7^-bound peptide substrate. (**a**) Schematic showing assembly of steady-state ubiquitylation reactions. The indicated protein components are first incubated in two separate tubes followed by mixing to initiate the reaction. After quenching, reactions are divided equally with one sample being treated with sodium hydroxide (NaOH). (**b**) Steady-state ubiquitylation assay containing wild-type (WT) cycE peptide substrate. S0 represents an unmodified peptide substrate, S1 is a substrate modified with a single ubiquitin (product), etc. WT or histidine-tagged no Lys (K0) ubiquitin proteins were included as shown (the His-tag explains why ubiquitin conjugates migrate slightly more slowly than with WT ubiquitin). The peptide sequence is shown, where the phosphorylated Thr and Ser residues (orange) form the diphosphodegron motif that promotes binding to the CRL. Substrate Lys and Ser residues that in principle may serve as ubiquitin acceptors are green. The N-termini of all peptides have been acetylated (Ac) to block potential ubiquitylation of the amino group. All peptides have a C-terminal ‘GRRASY’ amino acid motif to enable ^32^P-labeling by protein kinase A. (**c**) Same as in (**b**) but with KR cycE peptide substrate. Mutated residues that eliminate the potential for ubiquitin modification are red. (**d**) Same as in (**c**) but with Lys only cycE peptide. (**e**) Same as in (**c**) but with Ser only cycE. (**f**) Graph showing quantification of product formation for the various reactions after a 60 s incubation period. Fraction ubiquitylated is defined as the ratio of the signal from all products divided by the sum of substrate (S0) and products. Representative autoradiograms are shown for *n* = 3 technical replicates.

Steady-state ubiquitylation reactions (Figure 2a) containing phospho(^32^P)-labeled WT cycE peptide, ARIH1, CRL1^FBXW7^ and WT ubiquitin led to robust ubiquitylation (Figure 2b). Next, oxyester bond formation between Ser residues and ubiquitin was probed by treatment of the samples with sodium hydroxide (NaOH). However, little difference was observed. Since the presence of WT ubiquitin led to poly-ubiquitin chains that confounded the detection of oxyester bonds, a ubiquitin mutant was used where all Lys acceptors had been mutated to Arg (K0 ubiquitin). These experiments resulted in the appearance of three distinct product bands (Figure 2b), with the intensities of the two slowest migrating products being greatly reduced upon treatment with NaOH (Figure 2b).

 Next, the KR cycE peptide was employed. Two product bands were observed with K0 ubiquitin (Figure 2c), and treatment with NaOH resulted in total loss of signal (Figure 2c,f). The NaOH treatment was specifically affecting oxyester bonds, since ubiquitylation of the Lys only cycE peptide resulted in the product but treatment with NaOH had no effect (Figure 2d,f). Importantly, product formation was exquisitely dependent on the presence of both the FBXW7-SKP1 substrate receptor and neddylated CUL1-RBX1 (Supplementary Figure S1a). ARIH1-catalyzed Ser ubiquitylation appears to be dependent on the position and/or the environment of the Ser residue, as employing the Ser only cycE peptide led to significantly less product than in comparison with KR cycE peptide (Figure 2e,f).

Two additional peptides were generated to further assay for non-canonical ubiquitylation. Replacing the N-terminal Ser residue with a Thr resulted in substantially lower efficiencies of product formation compared with the Ser only cycE peptide (Supplementary Figure S1b). We then assessed the efficiency of ARIH1-catalyzed ubiquitin transfer to an N-terminal amino group (N_amino_Arg only cycE). Product formation was robust but clearly weaker in comparison with the WT cycE peptide (Supplementary Figure S1c).

### CycE residue Ser 387 is the predominant site of non-canonical ubiquitylation

**Figure 3.**
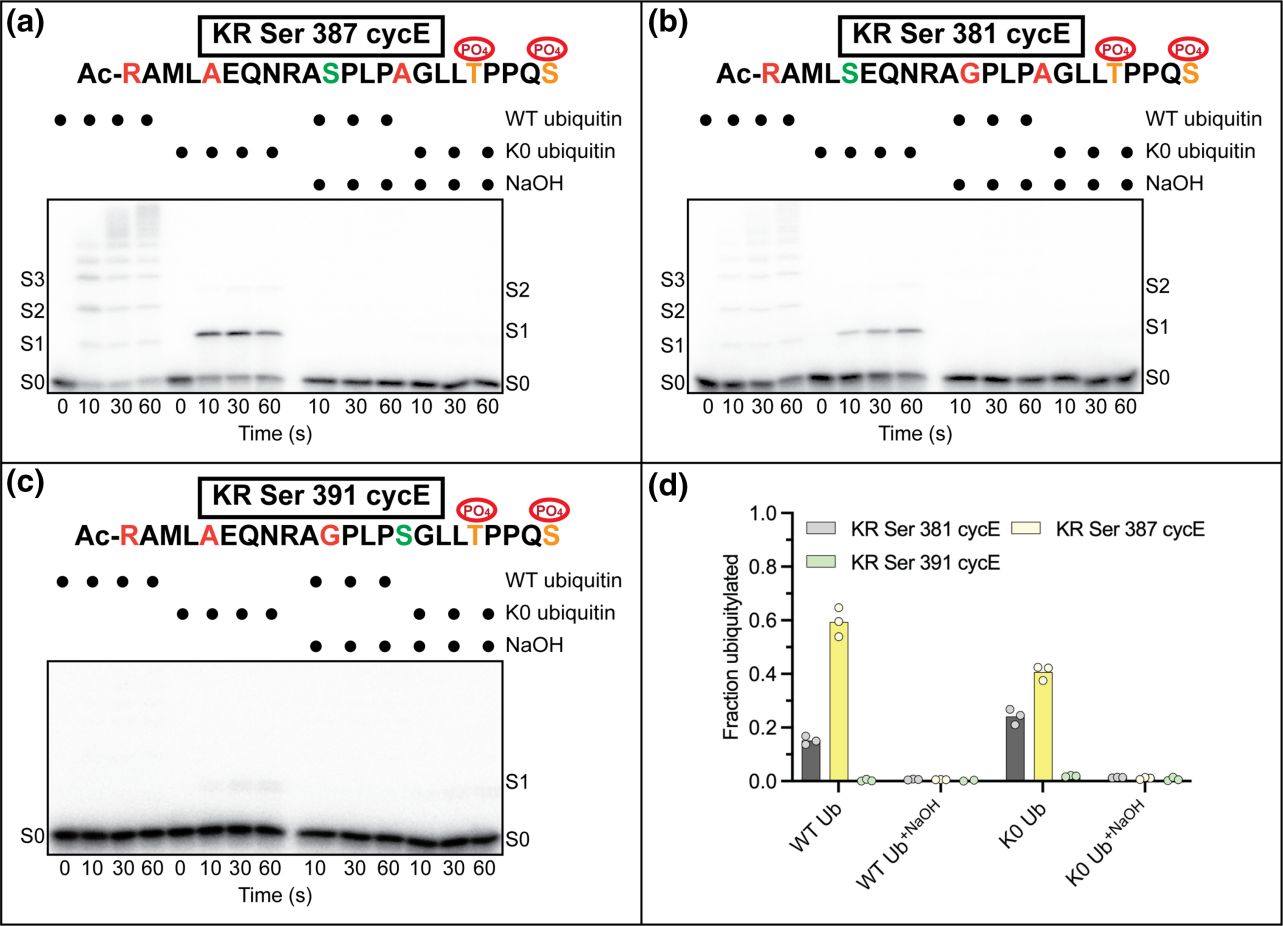
ARIH1 preferentially targets Ser 387 on KR cycE peptide substrate. (**a**) Steady-state ubiquitylation assay for KR Ser 387 cycE peptide. Despite the peptide containing a single Ser ubiquitin acceptor at position 387, notice how similar the pattern of ubiquitylation is with KR cycE (Figure 2c) that contained all three native Ser residues. The color scheme for the peptide is identical with that as described in Figure 2. (**b**) Same as in (**a**) except with KR Ser 381 cycE peptide. (**c**) Same as in (**a**) except with KR Ser 391 cycE peptide. (**d**) Graph showing quantification of the amounts of product formation after a 60 s incubation period for the three single Ser peptide substrates as shown. Fraction ubiquitylated is defined as the ratio of the signal from all products divided by the sum of substrate (S0) and products. Representative autoradiograms are shown for *n* = 3 technical replicates.

To uncover the identities of the Ser residues being ubiquitylated, three additional cycE peptides were generated that contained a single Ser residue (KR Ser 381, KR Ser 387 and KR Ser 391 cycE). Ubiquitylation of the KR Ser 387 cycE peptide was most efficient (Figure 3a,d), followed by KR Ser 381 cycE (Figure 3b,d), whereas KR Ser 391 cycE ubiquitylation was only weakly detectable (Figure 3c,d). The treatment of all samples with NaOH led to the complete hydrolysis of ubiquitin conjugates. We wondered whether the replacement of Ser 387 with a Lys residue as the single potential ubiquitin acceptor within the peptide would also result in robust ubiquitylation. Modification of KR Lys 387 cycE was similar compared with both WT and Lys only cycE peptides (Supplementary Figure S2).

### Ser 387 ubiquitylation is modestly slower in comparison with Lys 377

**Figure 4.**
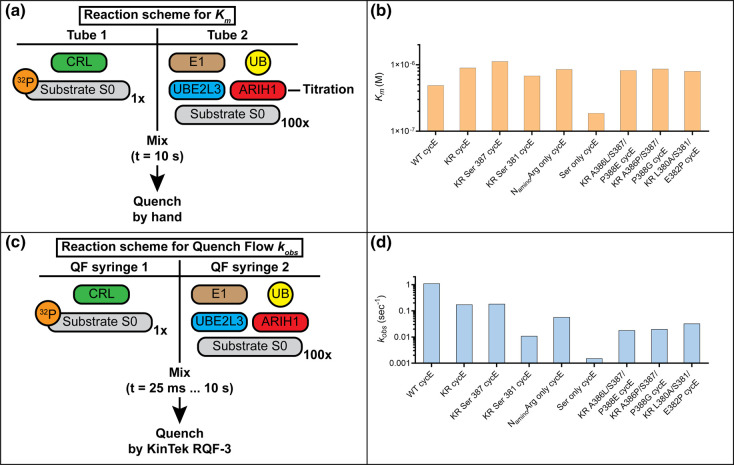
Ser 387 ubiquitylation is only modestly slower in comparison with Lys modification. (**a**) Schematic showing the assembly of single-encounter ubiquitylation reactions for estimation of the *K_m_* of ARIH1 for various CRL-substrate complexes. The titration of ARIH1 levels enables estimation of the *K_m_* by fitting how product formation varies with ARIH1 concentration to the Michaelis–Menten equation. (**b**) Graph showing the *K_m_* values of ARIH1 for CRL1^FBXW7^ and the cycE peptide substrates as shown. (**c**) Schematic showing the assembly of pre-steady-state kinetic ubiquitylation assays performed by quench flow on a Kintek RQF-3 instrument. The indicated proteins were pre-incubated prior to loading into the sample loops and initiation of the reaction by the control pad. Reactions were quenched with 2X SDS–PAGE loading buffer at various time points as early as 25 ms. Notice that these reactions were single-encounter between CRL and substrate: dissociation of radiolabeled substrate or product from the CRL likely results in their replacement with unlabeled substrate (S0) owing to its relatively high concentration (thus enabling estimation of the rates of ubiquitin transfer). (**d**) Same as (**b**), except for the rates of ubiquitin transfer, *k*_obs_. The standard errors of measurement for both *K_m_* and *k*_obs_ can be found in Table 1.

To quantitatively assess the rates of ARIH1-catalzyed isopeptide or oxyester bond formation, pre-steady-state kinetics were performed on a quench flow instrument. Prior to these experiments, the *K_m_* values of ARIH1 for CRL1^FBXW7^ were estimated (Figure 4a) to ensure that ARIH1 levels were always sufficient to approach saturation of the CRL complex. The *K_m_* values varied only modestly for all peptides (Table 1, Figure 4b and Supplementary Figure S3).

**Table 1.** Kinetic parameters for ARIH1 substrate priming

CRL	Substrate	*K_m_* (10^−6^ M)	*k*_obs_ (s^−1^)	*k*_obs_/*K_m_* (M^−1^s^−1^)	
SCF^FBXW7^	WT cycE	0.486 ± 0.092	1.12 ± 0.07	2.3 × 10^6^	
SCF^FBXW7^	KR cycE	0.895 ± 0.197	0.175 ± 0.014	2.0 × 10^5^	
SCF^FBXW7^	KR Ser 387 cycE	0.632 ± 0.119	0.183 ± 0.016	2.9 × 10^5^	
SCF^FBXW7^	KR Ser 381 cycE	0.681 ± 0.093	0.0109 ± 0.0003	1.6 × 10^4^	
SCF^FBXW7^	N_amino_ Arg only cycE	0.849 ± 0.124	0.0580 ± 0.0028	6.8 × 10^4^	
SCF^FBXW7^	Ser only cycE	0.187 ± 0.029	0.00152 ± 0.0002	8.2 × 10^3^	
SCF^FBXW7^	KR A386L/S387/P388E cycE	0.823 ± 0.232	0.0179 ± 0.0005	2.2 × 10^4^	
SCF^FBXW7^	KR A386P/S387/P388G cycE	0.864 ± 0.233	0.0200 ± 0.0005	2.3 × 10^4^	
SCF^FBXW7^	KR L380A/S381/E382P cycE	0.797 ± 0.185	0.0325 ± 0.0008	4.1 × 10^4^	
CUL2^VHL^	WT Hif1α	0.403 ± 0.061	0.367 ± 0.010	9.1 × 10^5^	
CUL2^VHL^	N_Ser_ Hif1α	0.660 ± 0.115	0.00244 ± 0.00010	3.7 × 10^3^	
CUL2^KLHDC2^	Lys47 Arg48 SelK	0.268 ± 0.050	0.0688 ± 0.004	2.6 × 10^5^	
CUL2^KLHDC2^	Arg47 Arg48 SelK	0.391 ± 0.109	0.0183 ± 0.0010	4.7 × 10^4^	
**ARIH1**	**CRL**	**cycE peptide**	***K_m_* (10^−6^ M)**	***k*_obs_ (s^−1^)**	**Fold change (*k*_obs_)**
WT	SCF^FBXW7^	KR Lys 387	0.726 ± 0.095	1.15 ± 0.08	—
WT	SCF^FBXW7^	Lys only	0.345 ± 0.068	0.983 ± 0.055	—
K342A/E343A	SCF^FBXW7^	Lys only	1.22 ± 0.15	0.0910 ± 0.0049	11
K342A/E343A	SCF^FBXW7^	KR Ser 387	0.461 ± 0.046	0.00491 ± 0.0002	37
K342A/E343A	SCF^FBXW7^	KR Lys 387	1.56 ± 0.32	0.0387 ± 0.0019	30
N358A	SCF^FBXW7^	Lys only	0.980 ± 0.164	0.225 ± 0.003	4
N358A	SCF^FBXW7^	KR Ser 387	1.04 ± 0.21	0.0159 ± 0.0006	12
N358A	SCF^FBXW7^	KR Lys 387	0.874 ± 0.130	0.145 ± 0.006	8
A397D/K398I/A399V	SCF^FBXW7^	Lys only	0.544 ± 0.065	0.536 ± 0.015	2
A397D/K398I/A399V	SCF^FBXW7^	KR Ser 387	1.19 ± 0.17	0.00708 ± 0.0003	26
A397D/K398I/A399V	SCF^FBXW7^	KR Lys 387	1.12 ± 0.21	0.0525 ± 0.0024	22

NA, no activity; *k*_obs_*/K_m_* is the catalytic efficiency with units M^−1 ^s^−1^; Fold change (*k*_obs_), the ratio of the WT and ARIH1 mutant *k*_obs_ values for the indicated peptide.

The rate of ubiquitin transfer from ARIH1 to CRL1^FBXW7^-bound WT cycE peptide was 1.12 s^−1^ (Table 1, Figure 4c,d and Supplementary Figure S4a), whereas the rate for KR cycE peptide (0.175 s^−1^) was only 6-fold slower (Table 1, Figure 4d and Supplementary Figure S4b). Interestingly, despite the KR cycE peptide having all three native Ser residues intact, a single product band was observed, indicating that ARIH1-mediated ubiquitin transfer was partitioning to a single Ser residue. Consistently, the rate of ubiquitin transfer from ARIH1 to KR Ser 387 cycE peptide was similar with the rate of KR cycE ubiquitylation (Table 1, Figure 4d and Supplementary Figure S4c). However, the rate of ubiquitin transfer to KR Ser 381 cycE was 18-fold slower (Supplementary Figure S4d), and product formation with KR Ser 391 cycE peptide was too weak to enable quantitative kinetics. Notably, the rate of ubiquitylation of an N-terminal amino group (N_amino_Arg only cycE) was 3-fold slower than the rate of oxyester formation to Ser 387 (Table 1, Figure 4d and Supplementary Figure S4e).

We next probed for the impact of changing the Ser position and/or environment on ARIH1-mediated oxyester ubiquitylation. Remarkably, the rate for Ser only cycE was 120-fold slower in comparison with KR Ser 387 peptide (Table 1, Figure 4d and Supplementary Figure S4f). To more systematically explore how position and environment may affect ARIH1 activity, additional cycE peptides were generated (Supplementary Figure S5a). First, KR Ser 387 cycE was modified where residues occupying positions 386 and 388 were replaced with those immediately surrounding either Ser 381 or Ser 391 in the WT peptide (Supplementary Figure S5b–e). These changes both resulted in order-of-magnitude decreases in the rates of ubiquitin transfer (Table 1 and Figure 4d). Next, KR Ser 381 cycE was modified such that residues 380 and 382 were replaced with those occupying positions 386 and 388 in the WT peptide (Supplementary Figure S5f,g). Interestingly, this resulted in a 3-fold increase in the rate of ubiquitin transfer (Table 1 and Figure 4d), whereas the same modification to KR Ser 391 cycE appeared to have little or no effect on product formation (Supplementary Figure S5h). Thus, it appears that, while the position of the Ser residue was shown to affect the efficiency of ubiquitylation, the environment of the surrounding residues was also an important factor that determines the rate of oxyester ubiquitylation.

### ARIH1 residues direct ubiquitylation of hydroxy and amino groups at position 387

**Figure 5.**
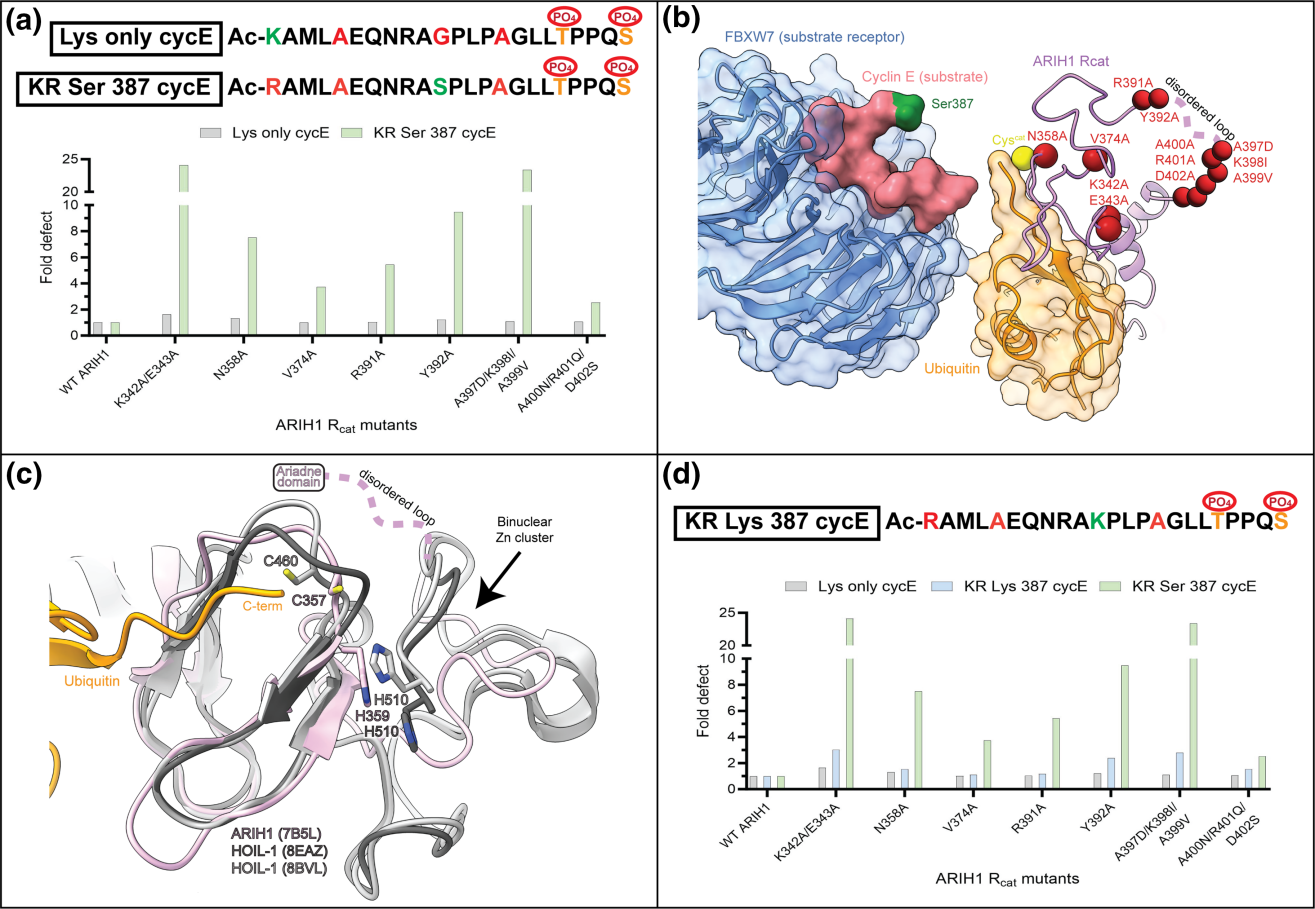
Identification of ARIH1 Rcat domain residues that promote ubiquitylation of acceptors at cycE peptide position 387. (**a**) Graph showing the fold defects of selected Rcat mutant proteins for isopeptide bond formation with Lys only cycE (gray) or KR Ser 387 cycE (light green) peptides. Fold defect is defined as the ratio of product formation by WT ARIH1 to that for mutant protein using the single-encounter assay (also see Supplementary Figure S6 and Excel File S1). (**b**) Ribbon diagram of a structural model from cryo-EM of a stable proxy for ARIH1 ∼ ubiquitin bound to a neddylated CRL1^FBXW7^-cycE peptide complex (EMD-12039). Surface representation is shown for FBXW7, cycE peptide and donor ubiquitin subunits. The locations of residues that may function preferentially in Ser ubiquitylation are shown as red spheres. The largest cluster of such residues was located at a region that lacked electron density (391–402). (**c**) Superposition of the ARIH1 Rcat domain (light-pink) with two recently determined HOIL-1 X-ray structures (light- and dark-gray). The positions of the catalytic Cys and His residues are shown. Notice that the region identified as most important for position-dependent ubiquitylation is located adjacent to a HOIL-1 binuclear zinc cluster thought to be involved in substrate binding. (**d**) Same as (**a**) except including results with the KR Lys 387 cycE peptide (light blue).

The chemistry for the catalysis of oxyester bond formation is unique in comparison with isopeptides since nucleophile activation involves far greater suppression of the *pK_a_* value by the enzyme (see Discussion). A comprehensive panel of ARIH1 mutants in the Rcat domain [42] was employed to determine the effects of mutation on the efficiencies of Lys only cycE ubiquitylation (initially chosen since Lys 377 is the physiological site of CYCLIN E ubiquitylation in cells) in comparison with KR Ser 387 cycE peptide. Product formation was first quantified for both peptides (Supplementary Figure S6 and Excel File S1) followed by comparison of mutants with WT ARIH1 (Figure 5a).

Mapping of the locations of residues whose mutation preferentially affected Ser ubiquitylation onto the structure of a stable proxy for ARIH1 ∼ ubiquitin bound to CRL1^FBXW7^ and cycE peptide [32] highlighted multiple secondary structure elements in proximity to the catalytic Cys (Figure 5b). A contiguous stretch of residues from Arg 391 to Asp 402, located at the boundaries of the Rcat and Ariadne domains, contained the highest concentration of residues shown to selectively participate in Ser ubiquitylation. While these residues appeared to be disordered in the CRL1^FBXW7^ cryo-EM structure, the ARIH1 residues that immediately precede the loop were located where the ARIH1 and HOIL-1 Rcat structures diverge from each other (Figure 5c; see Discussion).

One weakness of the Rcat mutant results is that, while differences in activity may be caused by a selective effect on isopeptide or oxyester bond formation, the distinct positions and environments of the ubiquitin acceptor site on cycE peptide (Lys 377 and Ser 387, respectively) may also affect the outcome. To differentiate between these two possibilities, the Rcat mutant screen was reemployed for ubiquitylation of the KR Lys 387 cycE peptide, enabling direct comparison with KR Ser 387 cycE (Supplementary Figure S7 and Excel File S1). Remarkably, several mutants now displayed significant defects in isopeptide bond formation that had shown mild or even negligible effects with the Lys only cycE peptide (Figure 5d).

Since the Rcat mutant biochemical screen employed a single 10 s timepoint, and with WT ARIH1-catalyzed ubiquitin transfer often occurring on the millisecond time scale, it is possible that even greater defects in Rcat mutant ARIH1 activity may be revealed upon quench flow. Thus, pre-steady-state kinetics were performed on Rcat mutants that displayed defects in Ser 387 and Lys 387 ubiquitylation but were less defective for Lys 377 modification. While the A397D/K398I/A399V ARIH1 mutant was only 2-fold defective in isopeptide bond formation to Lys 377 in comparison with WT ARIH1 (Supplementary Figure S8a,b), oxyester bond formation to Ser 387 was 26-fold defective (Table 1 and Supplementary Figure S9a,b). However, the rate of ubiquitin transfer to Lys 387 was nearly as defective as for KR Ser 387 cycE ubiquitylation (Supplementary Figure 9c,d), despite the Lys only and KR Lys 387 cycE peptides displaying nearly identical rates in the presence of WT ARIH1 (Table 1 and Supplementary Figures S8c,d and S9e,f). Similar results were obtained for K342A/E343A and N358A ARIH1 mutants (Supplementary Figures S10 and S11, respectively). In summary, several ARIH1 residues located within the Rcat domain appear to affect the modification of both amino and hydroxy ubiquitin acceptor groups when located at position 387 in the cycE peptide.

### ARIH1 participates in Ser ubiquitylation of CRL2-bound peptide substrates

**Figure 6.**
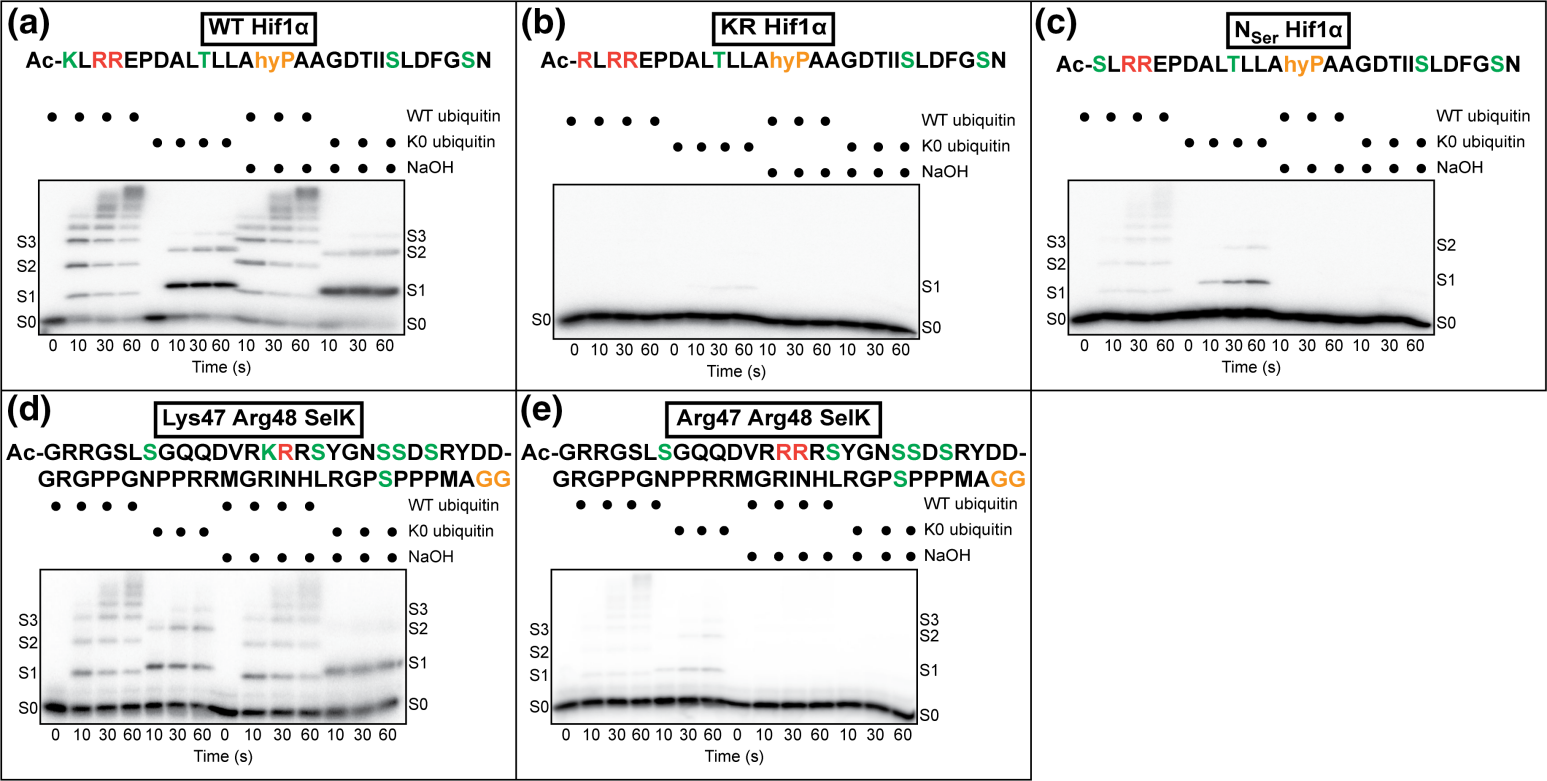
Ser ubiquitylation can occur on CRL2 substrates. (**a**) Steady-state ubiquitylation assay containing WT Hif1α peptide substrate. The color scheme for the peptide is identical with that as described in Figure 2, with the exception that the degron contains a hydroxylated Pro residue. (**b**) Same as in (**a**) but with KR Hif1α peptide. (**c**) Same as in (**a**) but with N_ser_ Hif1α peptide. Experiments in (**a**)–(**c**) were performed with neddylated CUL2-RBX1 in complex with the VHL-ELONGIN B/C substrate receptor. All peptides contained a C-terminal GRRASY motif for labeling with ^32^P. (**d**) Steady-state ubiquitylation assay containing SelK peptide. Since the degron includes the C-terminal region, an N-terminal GRRGSL protein kinase A labeling site was employed instead of the C-terminal one. Lys 48 was replaced with Arg to generate the single Lys substrate Lys47 Arg48 SelK. (**e**) Same as (**d**) except with Arg47 Arg48 SelK. Experiments in (**d**) and (**e**) were performed with neddylated CUL2-RBX1 in complex with the KLHDC2-ELONGIN B/C substrate receptor. Autoradiograms are representative of *n* = 3 technical replicates.

We next explored whether CRL-dependent substrate Ser ubiquitylation by ARIH1 may occur beyond CRL1^FBXW7^ and cycE peptide. Two additional *in vitro* reconstituted CRL ubiquitylation systems were employed: a neddylated CRL containing the cullin subunit CUL2 with the substrate receptor VHL-ELONGIN B/C (CRL2^VHL^) and Hif1α peptide substrate [44,45] and a second CRL2 in complex with the substrate receptor KLHDC2-ELONGIN B/C (CRL2^KLHDC2^) and SelK peptide substrate [46,47]. Both peptides were based on the native amino acid sequences of the full-length proteins and contained multiple Ser and/or Thr residues (Figure 6a,d).

While robust poly-ubiquitin chain formation was apparent with the WT Hif1α peptide in the steady-state assay, there was no detectable change after exposure to NaOH (Figure 6a). A modified Hif1α peptide was then synthesized where the sole Lys residue had been replaced with an Arg (KR Hif1α). While product formation was extremely weak, NaOH treatment nevertheless resulted in the disappearance of the signal (Figure 6b). Finally, a Hif1α peptide was prepared where the N-terminal Lys residue was replaced with a Ser (N_Ser_ Hif1α). Here product formation was observed, susceptible to NaOH (Figure 6c), and the amount of product was comparable to Ser only cycE. Quantitative kinetics were performed and showed approximately 100-fold greater activity of ARIH1 with the WT Hif1α peptide in comparison with N_Ser_ Hif1α (Table 1 and Supplementary Figure S12). Ubiquitylation of SelK containing a single Lys (Lys47 Arg48 SelK) by ARIH1 and CRL2^KLHDC2^ was apparent in the steady-state assay, and a peptide where the Lys had been replaced with Arg (Arg47 Arg48 SelK) displayed ubiquitylation of as many as three Ser residues (Figure 6d,e). On the other hand, the rates of ubiquitin transfer from ARIH1 to both SelK peptides were slow in comparison with WT and KR cycE (Table 1 and Supplementary Figure S13).

## Discussion

The ubiquitylation of proteins on non-canonical residues is an emerging field. Here we identify Ser ubiquitylation promoted by the RBR E3 ARIH1, where: (1) ARIH1 appears to display specificity for the ubiquitylation of a Ser residue in cycE peptide; (2) the efficiency of ubiquitylation is dependent on the chemical environment surrounding the Ser; (3) the fastest rate of Ser ubiquitylation was only modestly slower than ARIH1-catalyzed Lys modification; and (4) ARIH1 residues were identified that affect both Ser and Lys ubiquitylation, especially when the acceptor group resided at position 387 within the cycE peptide.

At least two factors may be responsible for ARIH1's specificity for Ser 387 on cycE peptide substrate compared with Ser 381 and Ser 391. First, the span between the phosphodegron motif on substrate that promotes binding to the CRL and the ubiquitin acceptor residue appears to be important. Notably, this was not a factor for WT ARIH1-catalyzed isopeptide bond formation here (Supplementary Figures S2, S9e,f and Table 1) or to various Lys residues on cycE-based peptides in a previous study [32]. A second factor was shown to be the residues that are immediately distal and proximal to the Ser residue being ubiquitylated. For instance, mutating the residues immediately adjacent to Ser 387 to those next to Ser 381 or Ser 391 resulted in greater than order-of-magnitude losses of activity, likely by affecting the local chemical environment surrounding the Ser side-chain hydroxy group.

Activation of the nucleophile that attacks the E3 ∼ ubiquitin thioester bond is an obligate step during ubiquitylation. Lys residues and their side-chain amino group typically have *pK_a_* values of approximately 10–11 in water, whereas hydroxy groups emanating from a Ser or Thr residue have *pK_a_* values of approximately 16 in similar environments. Studies have shown that ubiquitylating enzymes manipulate the local environments near the amino group to suppress the *pK_a_* value closer to physiological pH [48]. Given the far greater change in *pK_a_* necessary to promote oxyester bond ubiquitylation, E3s may have evolved unique strategies to promote this type of catalysis.

The possibility of catalysis or perhaps substrate binding being at least partially distinct in non-canonical ubiquitylation motivated a search for ARIH1 residues that differentially affect Ser ubiquitylation relative to Lys. Several residues within a loop (391–402) located at the C-terminal end of the Rcat domain were identified as being particularly important for oxyester bond formation in comparison with Lys 377 ubiquitylation. Surprisingly, the most defective mutant, A397D/K398I/A399V ARIH1 (whose mutations were chosen based on the residues at equivalent positions in the paralog ARIH2), was nearly as defective at isopeptide bond formation in comparison with Ser 387 ubiquitylation, but only when the Lys residue was located at position 387 within the cycE peptide. A similar pattern was observed for other ARIH1 Rcat mutants whose affected residues were located at sites other than the disordered loop, raising the possibility that their functional role may at least in part involve substrate alignment within the ARIH1 active site (Table 1).

Unfortunately, electron density for the ARIH1 391–402 loop was absent from a cryo-EM map corresponding to ARIH1 ∼ ubiquitin bound to a neddylated CRL complex [32]. However, recent structures of HOIL-1 suggested substrate binding roles for Rcat regions that are structurally adjacent to ARIH1 residues immediately upstream of the disordered loop (Figure 5c) [24,31,49,50]. Furthermore, experiments performed on ARIH1 bound to the 4EHP protein substrate in the absence of a CRL indicated the involvement of C-terminal Rcat residues including Trp 386 towards substrate binding [51]. Structural studies are necessary to further dissect how the ARIH1 loop participates in the ubiquitylation reaction.

### These observations may reflect only the beginning of an era of non-canonical ubiquitylation discovery

A survey of the literature describing non-canonical ubiquitylation suggests unanimity regarding the difficulty of detecting oxyester bonds between biological molecules and ubiquitin in cells [19,23]. This is due to a host of reasons, such as the relative instability of an oxyester bond that is susceptible to hydrolysis, especially under denaturing conditions, in comparison with canonical ubiquitylation. New techniques will need to be developed to inquire whether ARIH1 can catalyze non-canonical ubiquitylation in cells. Meanwhile, biochemical characterizations are relatively feasible and may serve as a guide for future cellular explorations.

## Methods

### Protein expression and purification

All peptide substrates were either synthesized in-house at the Max Planck Institute für Biochemie or purchased (Biosynth) at purities of at least 95%. All proteins are of human origin. WT ubiquitin was purchased as a lyophilized powder (R&D systems). K0 ubiquitin that contained an N-terminal 6-Histidine tag and where all Lys residues had been mutated to Arg was purified as described [52]. Fluorescently labeled ubiquitin was prepared as previously described [53]. Ubiquitin-activating enzyme E1 (UBA1) was expressed in *Trichoplusiani* High-Five insect cells and purified as described [54,55]. UBE2L3 and ARIH1 (WT and FEE mutant) were expressed in bacteria and purified as described [42]. UBE2R2 and UBE2D3 were expressed in bacteria and purified as described [56]. HOIL-1 was expressed in bacteria and purified as described [57]. CUL1-RBX1 complex was co-expressed in High-Five insect cells using baculoviruses for GST-TEV-RBX1 (5-C-term) and full-length CUL1 as described [58]. CUL2-RBX1 complex was similarly co-expressed in High-Five insect cells as described [47]. Neddylation of both CUL1-RBX1 and CUL2-RBX1 was accomplished as previously described [32,42,54]. SKP1-FBW7ΔD (a monomeric version of FBXW7 with N-terminal truncation at residue 263 to the C-terminus) was expressed in bacteria and purified as previously described [42] VHL-ELONGIN B/C complex was expressed in bacteria and purified as previously described [59] and a monomeric mutant version of KLHDC2-ELONGIN B/C complex was expressed in insect cells and purified as described [47].

### Peptide labeling

All peptide substrates were labeled with γ phosphate ^32^P ATP as follows. Labeling reactions containing 5 or 10 μM peptide, 16 μM ATP and protein kinase A in labeling buffer (New England Biolabs) were assembled and incubated at 30°C for 2 h. Occasionally, peptides were labeled at 50 μM upon which additional cold ATP (50 μM) was spiked into the reaction after 1 h to complete the phosphorylation of the entire population of peptides.

### E3 auto-ubiquitylation

Auto-ubiquitylation was performed under multi-turnover conditions for labeled ubiquitin and E3. The final concentrations of the reaction components were 0.3 μM E1, 2 μM UBE2L3 and 20 μM fluorescein-labeled WT ubiquitin in reaction buffer (30 mM HEPES, pH 7.8, 100 mM NaCl, 5 mM MgCl_2_, 2 mM ATP, 1 mM DTT and 50 μg/ml BSA). Reactions were initiated by adding ARIH1 FEE (a mutant version of ARIH1 that does not require neddylated CRL complex for activity) or HOIL-1 (2 μM final) and incubated (5 min at room temperature or 1 h at 37°C, respectively) prior to quenching with SDS–PAGE loading buffer (100 mM Tris–HCl, pH 6.8, 20% glycerol, 30 mM EDTA, 4% SDS and 4% β-mercaptoethanol). The quenched reactions were then split in half where samples were either incubated with water or 1.5 M sodium hydroxylamine for 20 min, 40 min and 60 min at 37°C. Samples were then diluted and immediately loaded onto NuPage 8–16% precast SDS–PAGE gels. Substrates and products were imaged using an Amersham Typhoon scanner and quantified using Amersham ImageQuant TL software. The signal for E3-ubiquitin was divided by the values of all labeled ubiquitin species in the lane followed by plotting of the data using GraphPad Prism software (version 8).

### Steady-state ubiquitylation assays

Ubiquitylation reactions were assembled into two separate mixtures by dilution of stock proteins into reaction buffer (30 mM Tris–HCl (pH 7.5), 100 mM NaCl, 5 mM MgCl_2_, 2 mM DTT and 2 mM ATP). For tube 1, either neddylated CUL1-RBX1 (1 μM) or neddylated CUL2-RBX1 (0.5 μM) were added followed by substrate receptor complex (FBXW7-SKP1 for CRL1 (1 μM) and VHL-ELONGIN B/C (0.5 μM) or KLHDC2-ELONGIN B/C (0.5 μM) for CRL2) followed by ^32^P-labeled peptide substrate (0.2 μM) and incubation for 15 min. Meanwhile, tube 2 was assembled by the addition of E1 enzyme (1 μM) and WT or K0 ubiquitin (12.5 μM) and was incubated for 1 min. Next, UBE2L3 was added (10 μM) and incubated for an additional 2 min to initiate the E2 charging reaction. Finally, ARIH1 (5 μM) was added and incubated for ∼1 min. After the final incubation period, the contents of tube 2 were aliquoted into three Eppendorf tubes that represent the time points of 10″, 30″ and 60″ followed by brief spinning in a microcentrifuge to collect the contents at the bottom of the tubes. Reactions were initiated by addition of an equal volume of tube 1 to each time point and then quenched in reducing 2× SDS–PAGE loading buffer (100 mM Tris–HCl (pH 6.8), 20% glycerol, 30 mM EDTA, 4% SDS and 4% β-mercaptoethanol) after the appropriate reaction time (see Figure 2a for a diagram of the reaction scheme).

To probe for the formation of oxyester bonds between peptide and ubiquitin, 2 μl of 1.5 M NaOH was added to 10 μl of each steady-state timepoint that had been quenched in 2× SDS–PAGE buffer (note that an equivalent volume of quenched reaction had been removed to assay for samples in the absence of NaOH). These mixtures were briefly collected by centrifugation and then incubated in a heating block for 1 h at 50°C. Next, the samples were briefly centrifuged to collect the formation of water condensates that had formed on the tube caps followed by the separation of substrates and products on 18% SDS–PAGE gels, autoradiography (Typhoon 5; Cytiva) and quantification (ImageQuant Cytiva).

### Estimation of the *K_m_* of ARIH1 for CRL-peptide substrate complexes

Ubiquitylation reactions were performed under single-encounter conditions with respect to the substrate and CRL complex. This is accomplished by first assembling a complex between the CRL and labeled substrate, followed by initiation of the reaction with ARIH1 ∼ ubiquitin in a solution containing 100-fold excess of the unlabeled peptide substrate. Thus, when labeled substrate and/or product dissociates from the CRL, reassociation is unlikely due to competition with cold peptide (see Figure 4a for a diagram of the reaction scheme).

Reaction components were assembled into two separate mixtures by dilution of stock proteins into reaction buffer as described in the steady-state ubiquitylation section. For tube 1, either neddylated CUL1-RBX1 (1 μM) or neddylated CUL2-RBX1 (0.5 μM) were added followed by substrate receptor complex (FBXW7-SKP1 for CRL1 (1 μM) and VHL-ELONGIN B/C (0.5 μM) or KLHDC2-ELONGIN B/C (0.5 μM) for CRL2) followed by ^32^P-labeled peptide substrate (0.2 μM) and incubation for 15 min. Meanwhile, a master mix of E1 (1 μM) and K0 ubiquitin (25 μM for CRL1^FBXW7^ and CRL2^VHL^-containing reactions and 50 μM for CRL2^KLHDC2^-containing reactions) was assembled (tube 2) and incubated for 1 min. Next, UBE2L3 protein was added (18.75 μM for CRL1^FBXW7^ and CRL2^VHL^ and 42.5 μM for CRL2^KLHDC2^) and incubated for two additional minutes. The contents of tube 2 were then evenly aliquoted (3 μl each) into nine individual Eppendorf tubes. Next, 1 μl of each concentration in a 2-fold dilution series of ARIH1 was added to each respective aliquot. Note that the UBE2L3 protein concentration was constant for all ARIH1 reactions in the titration series and always in excess of the ARIH1 concentration. All aliquots were then briefly centrifuged followed by the addition of 1 μl of excess unlabeled substrate (20 μM final in tube 2), and then briefly centrifuged once more. Note that since the off-rate of SelK peptide for CRL2^KLHDC2^ results in a half-life that is significantly slower than the time of incubation for the ubiquitylation reactions, single-encounter conditions were already apparent without the addition of cold competitor SelK peptide. Reactions were initiated by the addition of an equal volume of tube 1 into the tube 2 aliquots and then quenched in reducing 2× SDS–PAGE loading buffer after 10 s. Substrate and products were resolved on 18% SDS–PAGE gels followed by autoradiography using an Amersham Typhoon 5 imager (Cytiva) and quantification of substrate and product levels with ImageQuant software (Cytiva). The fraction of substrate converted to the ubiquitylated product of each reaction was estimated by determining the fraction of products (as defined as labeled-peptide substrate that had been modified by one or more ubiquitins) over the total signal comprising substrate and products. Fraction ubiquitylated was then plotted as a function of the ARIH1 concentration and the data were fit to the Michaelis–Menten equation using nonlinear curve fitting (GraphPad Prism 9 software).

### Estimation of the rate of ARIH1-catalyzed ubiquitin transfer, *k*_obs_, to CRL-peptide substrate complexes

Pre-steady-state ubiquitylation reactions were assembled as described in the steady-state ubiquitylation section with the following modifications (see Figure 4c for a diagram of the reaction scheme). Tube 2 contained E1 (1 μM), K0 ubiquitin (12.5 μM), UBE2L3 (10 μM), ARIH1 (5 μM) and unlabeled peptide (20 μM) except for reactions with SelK as described in the previous section. Following the incubation periods as described, the contents of tubes 1 and 2 were loaded into separate loops on a KinTek RQF-3 Quench-Flow instrument. Reactions were initiated by bringing the two mixes together using drive buffer (30 mM Tris–HCl (pH 7.5) and 100 mM NaCl) and then quenched at various time points in reducing 2× SDS–PAGE loading buffer. Substrate and products were resolved on 18% SDS–PAGE gels followed by autoradiography using an Amersham Typhoon 5 imager and quantification with ImageQuant software (Cytiva). Since the closed-form solutions for ARIH1-catalyzed priming are dependent on only the disappearance of substrate, S0 levels were quantified as the fraction of the total signal for each time point, and the rates of ubiquitin transfer were estimated by fitting to analytical closed-form solutions using Mathematica [43]. Reactions with CRL2^KLHDC2^ and Arg 47 Arg 48 SelK peptide were performed at the bench by hand and without excess unlabeled peptide owing to the relatively slow kinetics of product formation and dissociation of SelK from the CRL complex. The kinetics of SelK ubiquitylation were fit to a one-phase exponential growth model (GraphPad Prism 9 software).

### Single-encounter ubiquitylation reactions for the ARIH1 Rcat mutant biochemical screen

Reactions were performed as described in the steady-state ubiquitylation section except under single-encounter conditions with respect to labeled substrate for CRL (where the cold competitor peptide final concentration was 10 μM). For A397D/K398I/A399V ARIH1, the mutated residues were selected from the human ARIH1 paralog ARIH2 (aka TRIAD1) at equivalent positions in the primary structure.

## Data Availability

Data availability is not applicable to this manuscript owing to the nature of our data which are either fluorescence scans or autoradiograms that are provided here or within the Supplementary figures.

## Abbreviations

CRLs: cullin-RING E3 ligases
NaOH: sodium hydroxide
NH_2_OH: hydroxylamine
RBR: RING-in-between RING
UBA1: ubiquitin-activating enzyme E1
