# Supplementary material for: Catalysis of non-canonical protein ubiquitylation by the ARIH1 ubiquitin ligase
Source: Biochem J. 2023 Nov 17;480(22):1817–31. doi: 10.1042/BCJ20230373 (PMC10657180; doi:10.1042/BCJ20230373)
Supplement: Supplementary Material Table [file BCJ-480-1817-s1.pdf]

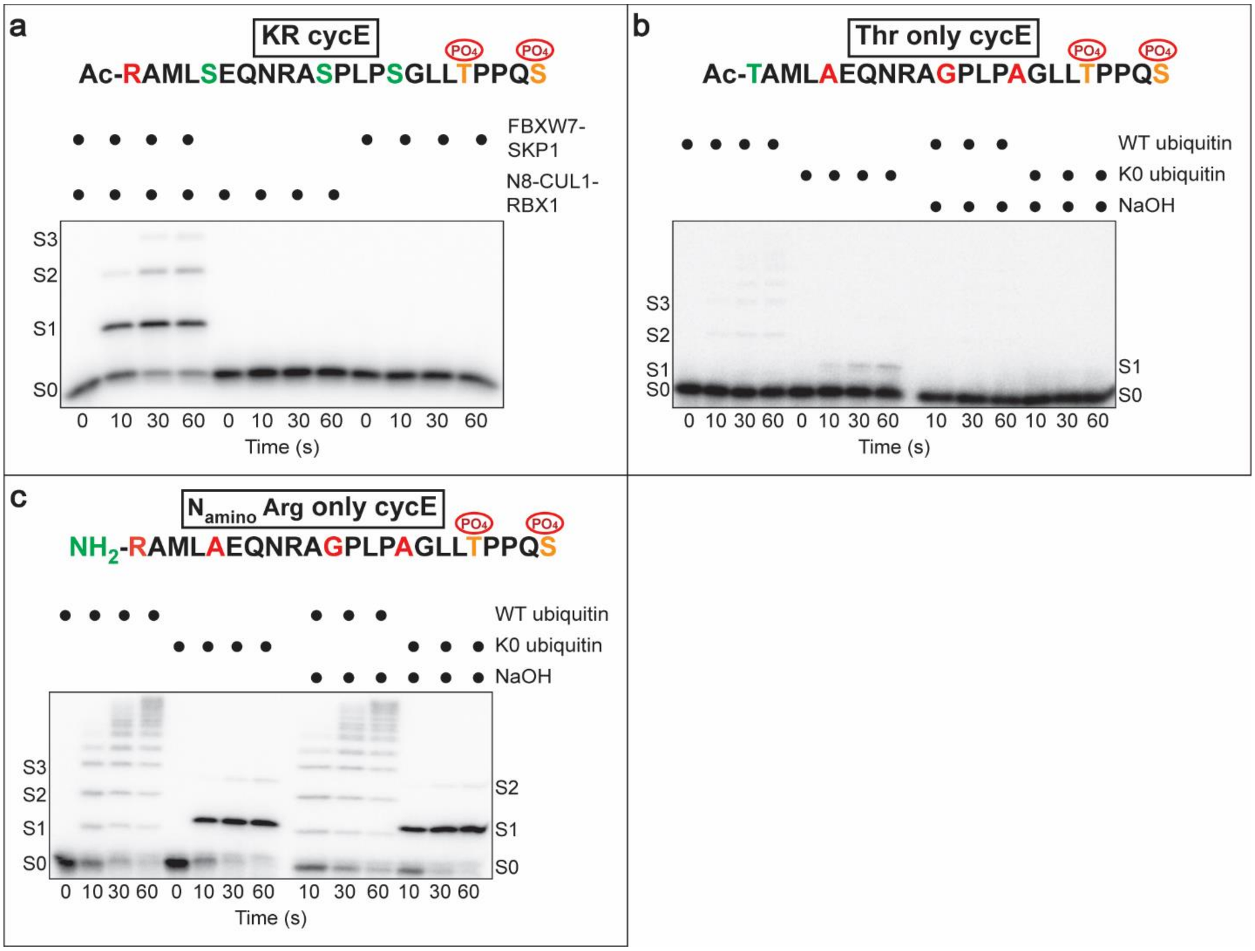

Supplementary Figure S1. Non-canonical ubiquitylation of a Thr residue or the N-terminal amino group on cycE peptide substrate. (a) Steady-state ubiquitylation reactions with KR cycE peptide. Product formation occurs only in the presence of both substrate receptor (FBXW7-SKP1) and neddylated CUL1-RBX1 (N8-CUL1-RBX1). (b) Steady-state ubiquitylation assay containing Thr only cycE peptide. (c) Same as in (b) but with N_{amino} Arg only cycE peptide. The color scheme for the peptides is identical to that as described in Figure 2. Representative autoradiograms are shown for n=2 (a) or n=3 technical replicates.

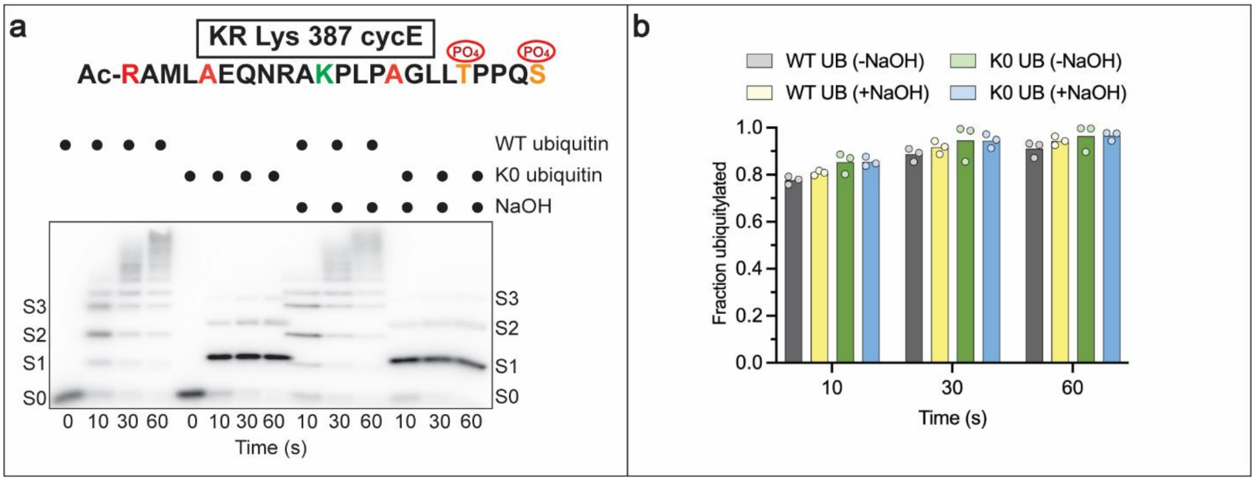

Supplementary Figure S2. Efficient ubiquitylation of a Lys residue at position 387 in the cycE peptide. (a) Steady-state ubiquitylation assay containing KR Lys 387 cycE peptide substrate. The color scheme for the peptide is identical to that as described in Figure 2. (b) Graphical representation of the fraction of ubiquitylated substrate (S0). Representative autoradiograms are shown for n=3 technical replicates.

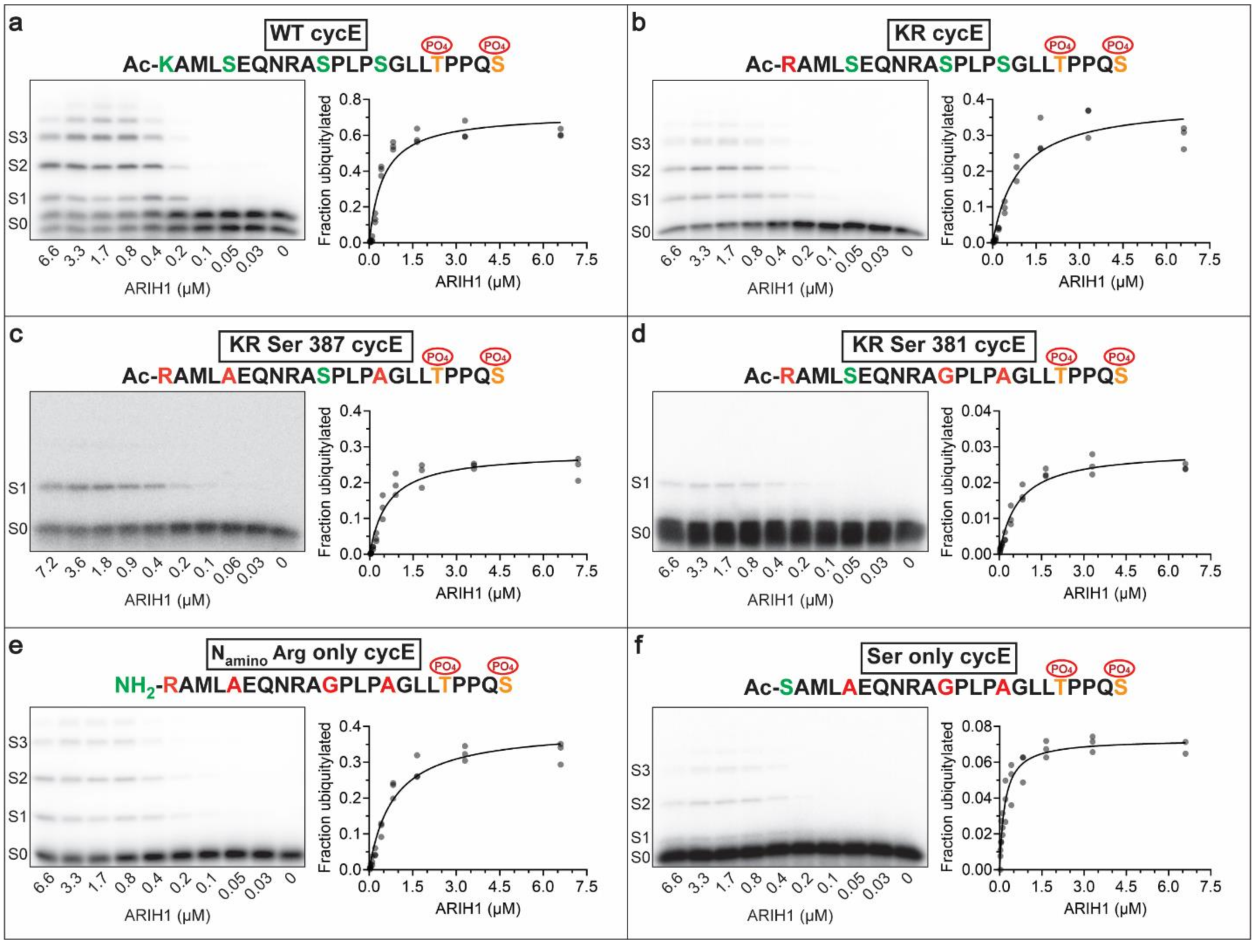

Supplementary Figure S3. Changing the cycE peptide composition only mildly affects the K_m of ARIH1 for CRL1^{FBXW7}. (a) Autoradiogram showing substrate (S0) conversion to product for ubiquitylation reactions containing increasing levels of ARIH1 (left) and the fit of the data to the Michaelis-Menten equation (right) with WT cycE peptide. (b) Same as (a) except with KR cycE peptide. (c) Same as (a) except with KR Ser 387 cycE peptide. (d) Same as (a) except with KR Ser 381 cycE peptide. (e) Same as (a) except with N_{amino} Arg only cycE peptide. (f) Same as (a) except with Ser only cycE peptide. The color scheme for the peptides is identical to that as described in Figure 2. Representative autoradiograms are shown for n=3 technical replicates.

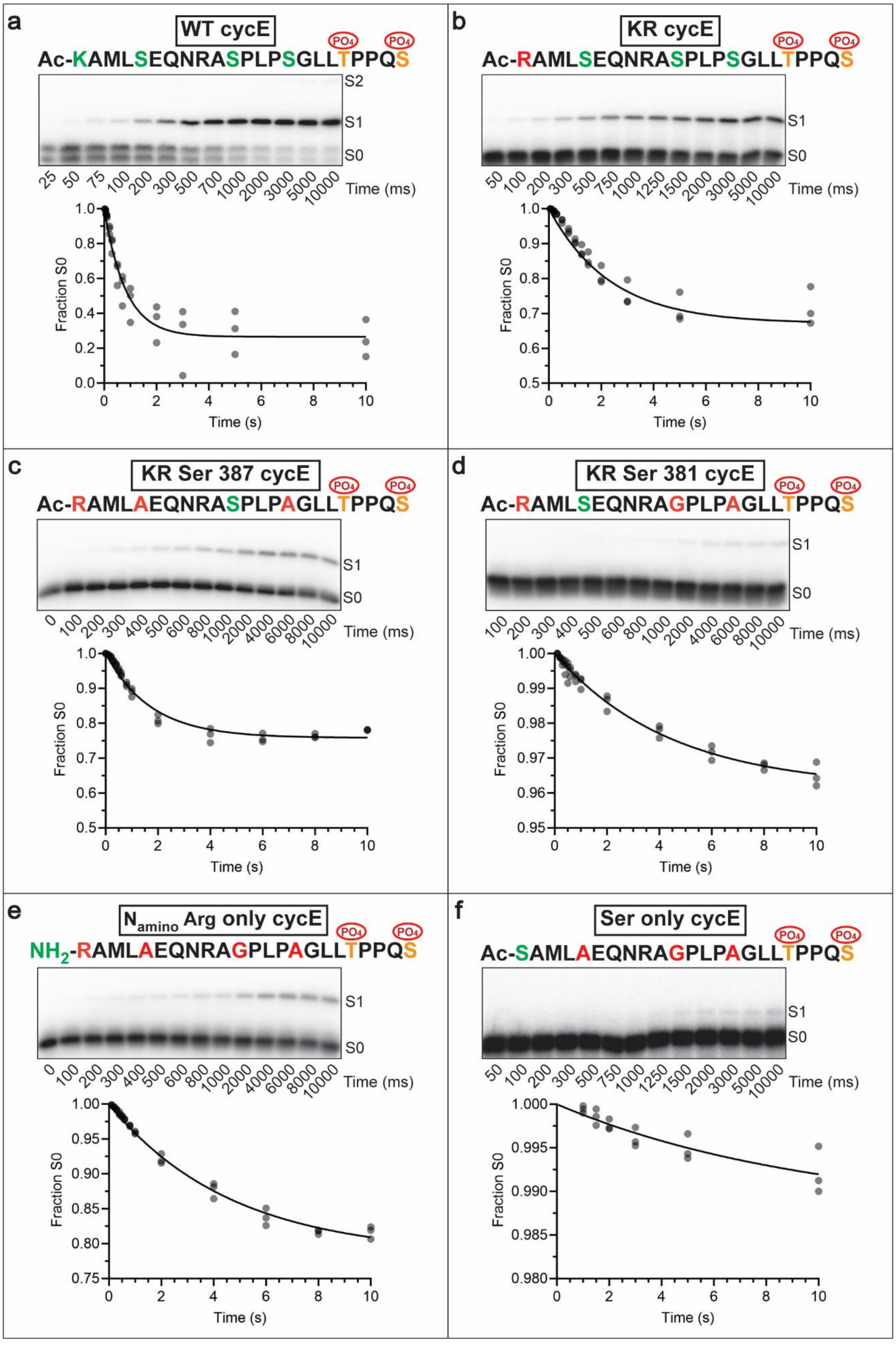

a
WT cycE
Ac-KAMLSEQNRASPLPSGLLTPPQS
PO4
S2
S1
S0
25 50 75 100 200 300 500 700 1000 2000 3000 5000 10000 Time (ms)
Fraction S0
Time (s)
b
KR cycE
Ac-RAMLSEQNRASPLPSGLLTPPQS
50 100 200 300 500 750 1000 1250 1500 2000 3000 5000 10000 Time (ms)
c
KR Ser 387 cycE
Ac-RAMLAEQNRASPLPAGLLTPPQS
0 100 200 300 400 500 600 800 1000 2000 4000 6000 8000 10000 Time (ms)
d
KR Ser 381 cycE
Ac-RAMLSEQNRAGPLPAGLLTPPQS
100 200 300 400 500 600 800 1000 2000 4000 6000 8000 10000 Time (ms)
e
N_{amino} Arg only cycE
NH_2-RAMLAEQNRAGPLPAGLLTPPQS
0 100 200 300 400 500 600 800 1000 2000 4000 6000 8000 10000 Time (ms)
f
Ser only cycE
Ac-SAMLAEQNRAGPLPAGLLTPPQS
50 100 200 300 500 750 1000 1250 1500 2000 3000 5000 10000 Time (ms)

Supplementary Figure S4. Estimation of the rate of ubiquitin transfer (*k_{obs}*) to various cycE peptide substrates. (a) Autoradiogram showing a time course for substrate (S0) conversion to product for pre-steady state ubiquitylation reactions (top) and the fit of the data to a closed form solution modeling the single-encounter reaction (bottom) with WT cycE peptide. (b) Same as (a) except with KR cycE peptide. (c) Same as (a) except with KR Ser 387 cycE peptide. (d) Same as (a) except with KR Ser 381 cycE peptide. (e) Same as (a) except with N_{amino} Arg only cycE peptide. (f) Same as (a) except with Ser only cycE peptide. The color scheme for the peptides is identical to that as described in Figure 2. Representative autoradiograms are shown for n=3 technical replicates.

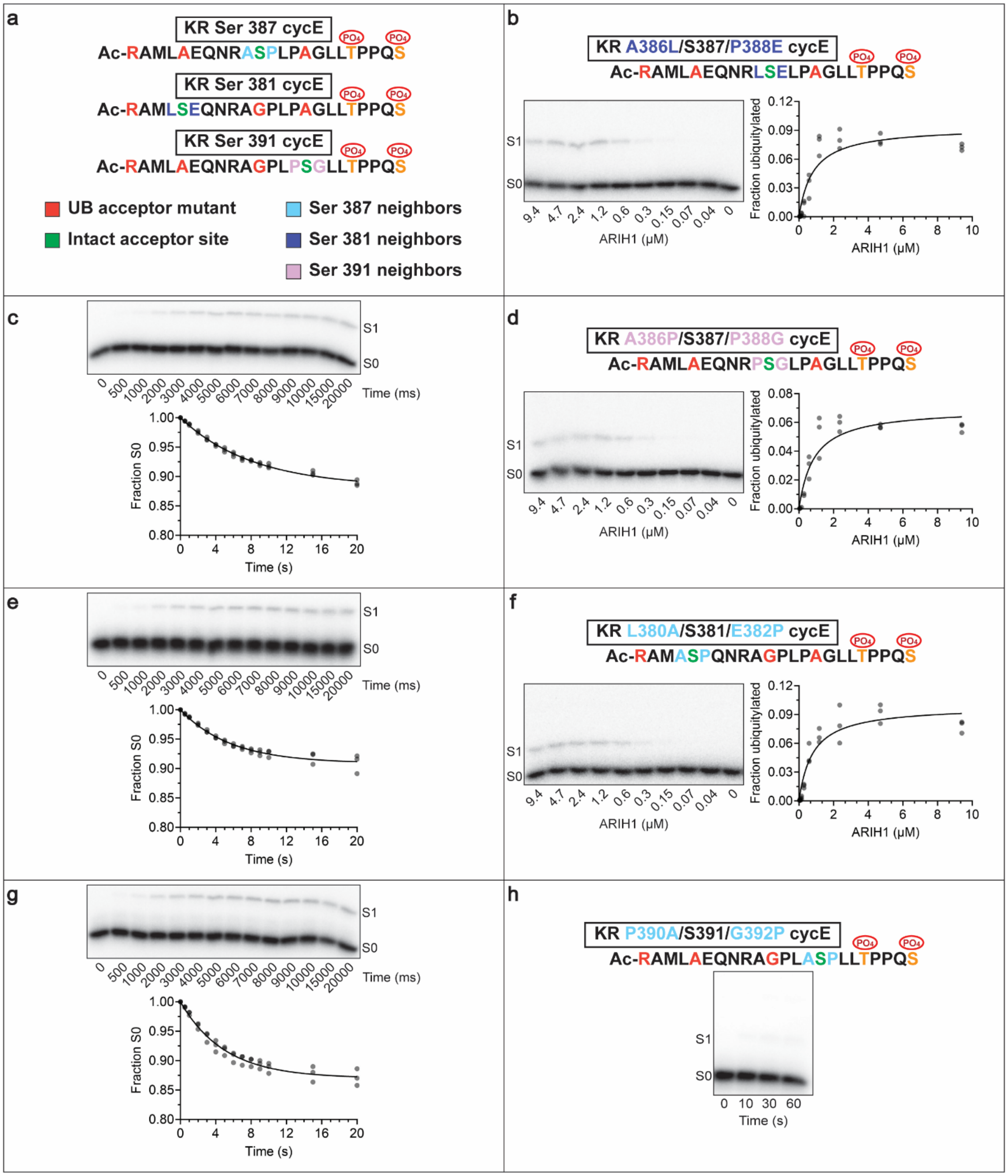

Supplementary Figure S5. The rate of ubiquitin transfer (k_{obs}) to Ser residues on cycE peptide is affected by their residue neighbors. (a) Peptide sequences for KR Ser 381, Ser 387 and Ser 391 cycE peptides. The color scheme has been explained in the Figure 2 legend, while additional colors are shown to highlight residue neighbors of each Ser. (b) Autoradiogram showing substrate (S0) conversion to product for ubiquitylation reactions containing increasing levels of ARIH1 (left) and the fit of the data to the Michaelis-Menten equation (right). The cycE

peptide contains a single Ser ubiquitin acceptor at position 387 and where the residues located at 386 and 388 have been replaced with those neighboring Ser 381. (c) Autoradiogram showing a time course for substrate (S0) conversion to product for pre-steady state ubiquitylation reactions (top) and the fit of the data to a closed form solution modeling the single-encounter reaction (bottom) with the peptide shown in (b). (d) Same as (b), except with a cycE peptide containing a single Ser at position 387 and replacement of residues located at 386 and 388 with those neighboring Ser 391. (e) Same as (c) except with the peptide shown in (d). (f) Same as (b), except with a cycE peptide containing a single Ser at position 381 and replacement of residues located at 380 and 382 with those neighboring Ser 387. (g) Same as (c) except with the peptide shown in (f). (h) Time course for a steady-state ubiquitylation reaction with cycE peptide that contained a single Ser at position 391 and replacement of residues located at 390 and 392 with those neighboring Ser 387. Representative autoradiograms are shown for n=3 technical replicates.

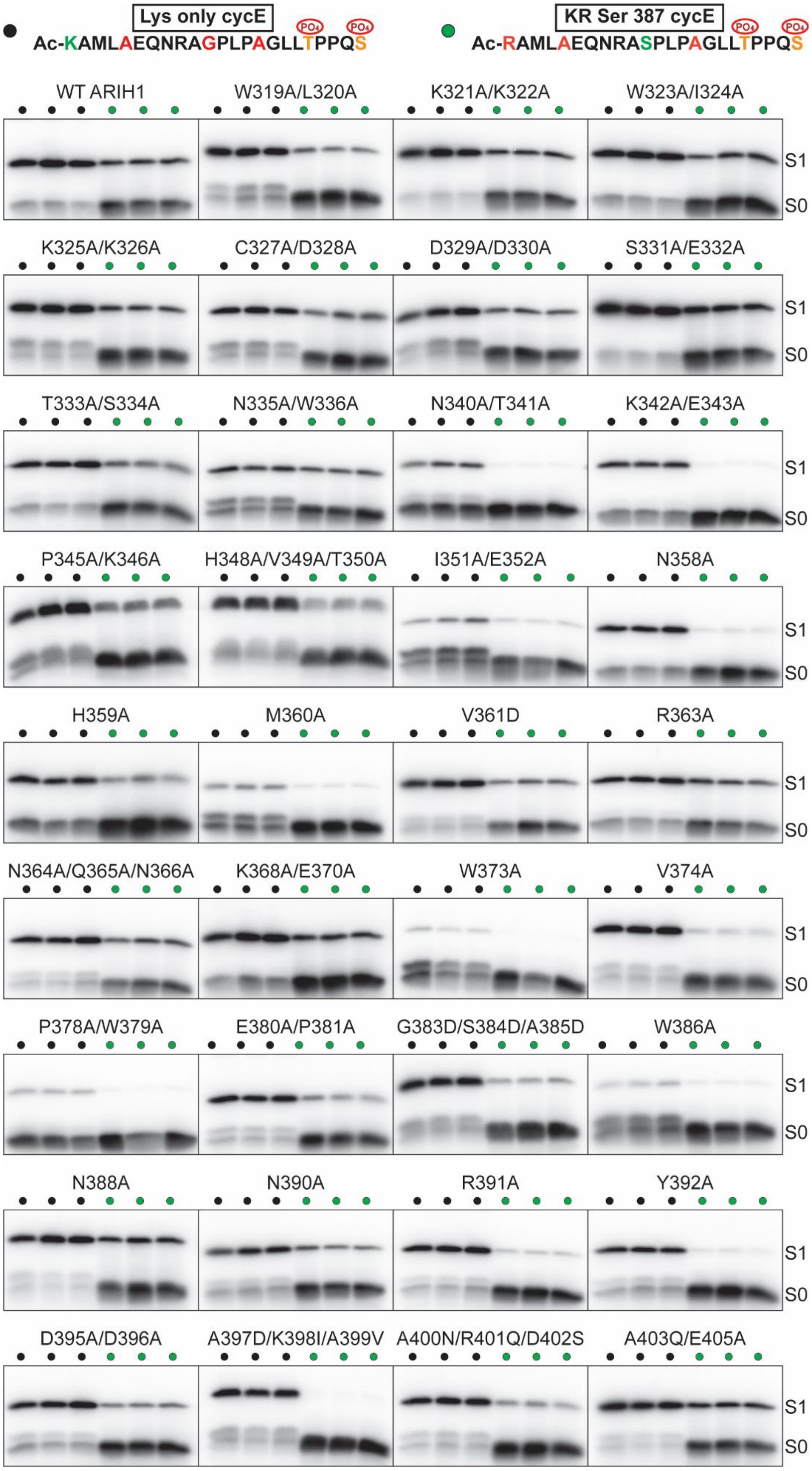

Lys only cycE
Ac-KAMLAEQNRAGPLPAGLLTPPQS
PO4
KR Ser 387 cycE
Ac-RAMLAEQNRASPLPAGLLTPPQS
WT ARIH1
W319A/L320A
K321A/K322A
W323A/I324A
K325A/K326A
C327A/D328A
D329A/D330A
S331A/E332A
T333A/S334A
N335A/W336A
N340A/T341A
K342A/E343A
P345A/K346A
H348A/V349A/T350A
I351A/E352A
N358A
H359A
M360A
V361D
R363A
N364A/Q365A/N366A
K368A/E370A
W373A
V374A
P378A/W379A
E380A/P381A
G383D/S384D/A385D
W386A
N388A
N390A
R391A
Y392A
D395A/D396A
A397D/K398I/A399V
A400N/R401Q/D402S
A403Q/E405A
S1
S0

Supplementary Figure S6. Ubiquitylation reactions identify ARIH1 Rcat mutant proteins that are selectively defective in Ser 387 modification compared with Lys 377. Autoradiograms showing triplicate technical data points for WT or mutant ARIH1-catalyzed ubiquitin transfer to Lys only cycE peptide (3 leftmost lanes) or to KR Ser 387 cycE (3 rightmost lanes) peptides. Unmodified substrate (S0) and ubiquitylated product (S1) are shown. Black dots above each lane represent ubiquitylation reactions containing the Lys only cycE peptide, while green dots represent reactions with KR Ser 387 cycE peptide.

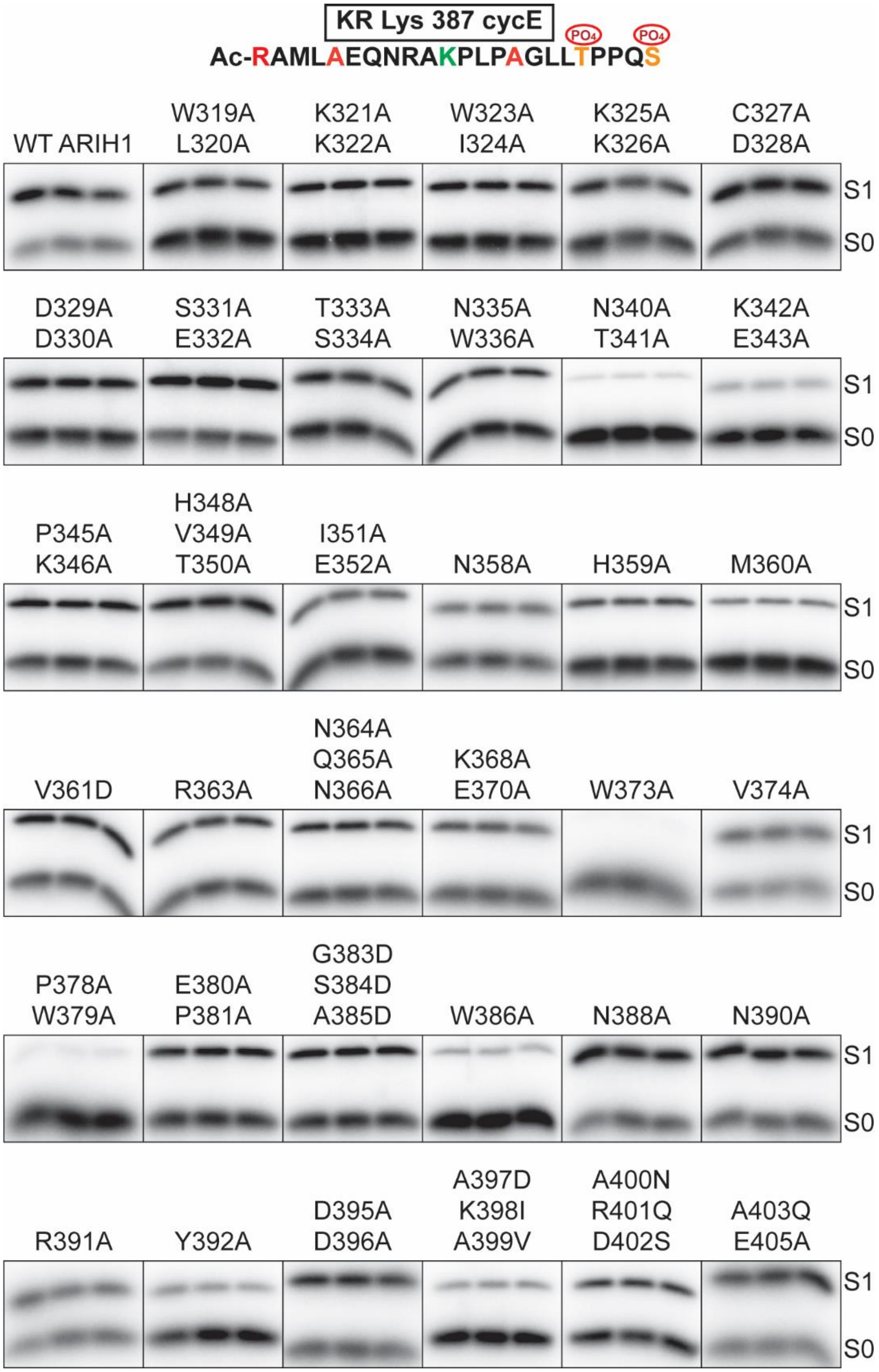

Supplementary Figure S7. Ubiquitylation assays reveal ARIH1 Rcat mutant proteins that are defective in Lys modification in a position-dependent manner. Autoradiograms showing triplicate technical data points for WT or mutant ARIH1-catalyzed ubiquitin transfer to KR Lys 387 cycE peptide. Unmodified substrate (S0) and ubiquitylated product (S1) are shown.

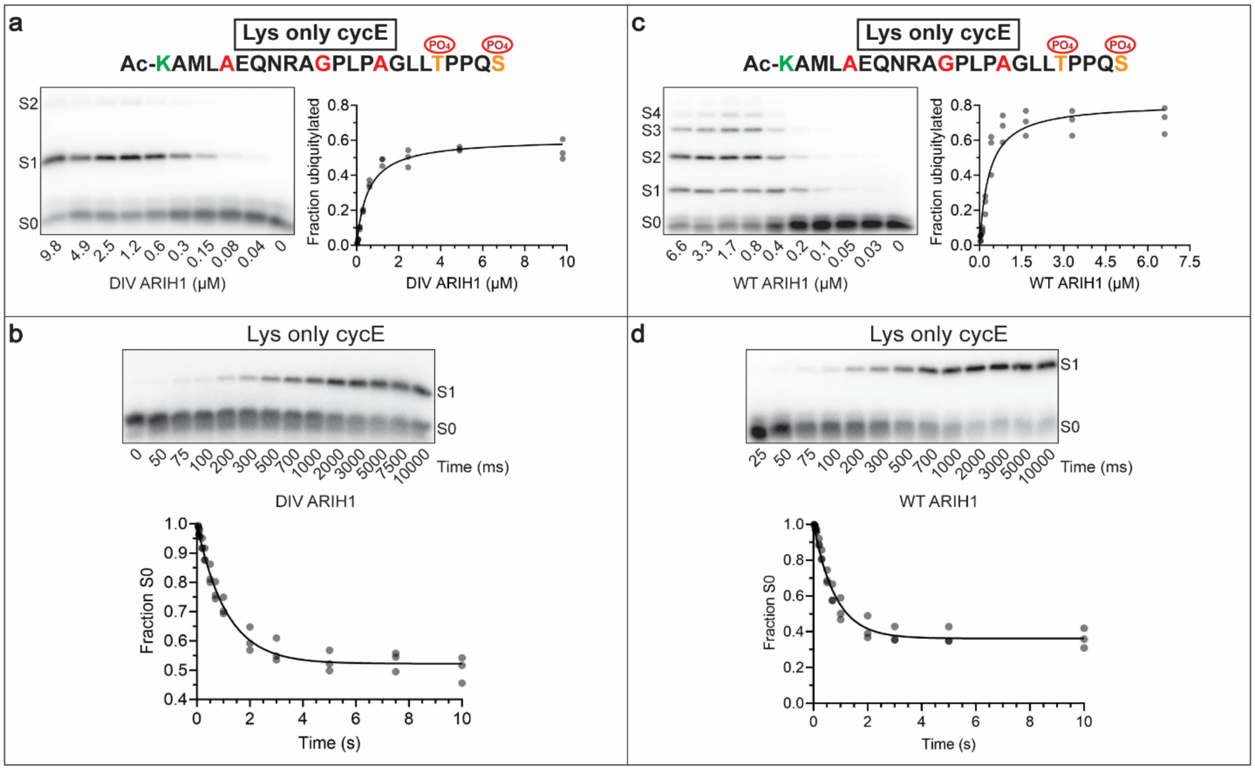

Supplementary Figure S8. Comparison of the kinetic parameters K_m and k_{obs} for WT and A397D/K398I/A399V 'DIV' ARIH1 with the Lys only cycE peptide. (a) Autoradiogram showing substrate (S0) conversion to product for ubiquitylation reactions containing increasing levels of DIV ARIH1 (left) and the fit of the data to the Michaelis-Menten equation (right) with Lys only cycE peptide. (b) Autoradiogram showing a time course for substrate (S0) conversion to product for pre-steady state ubiquitylation reactions (top) and the fit of the data to a closed form solution modeling the single-encounter reaction (bottom) with Lys only cycE peptide. (c) Same as (a) except with WT ARIH1. (d) Same as (b) except with WT ARIH1.

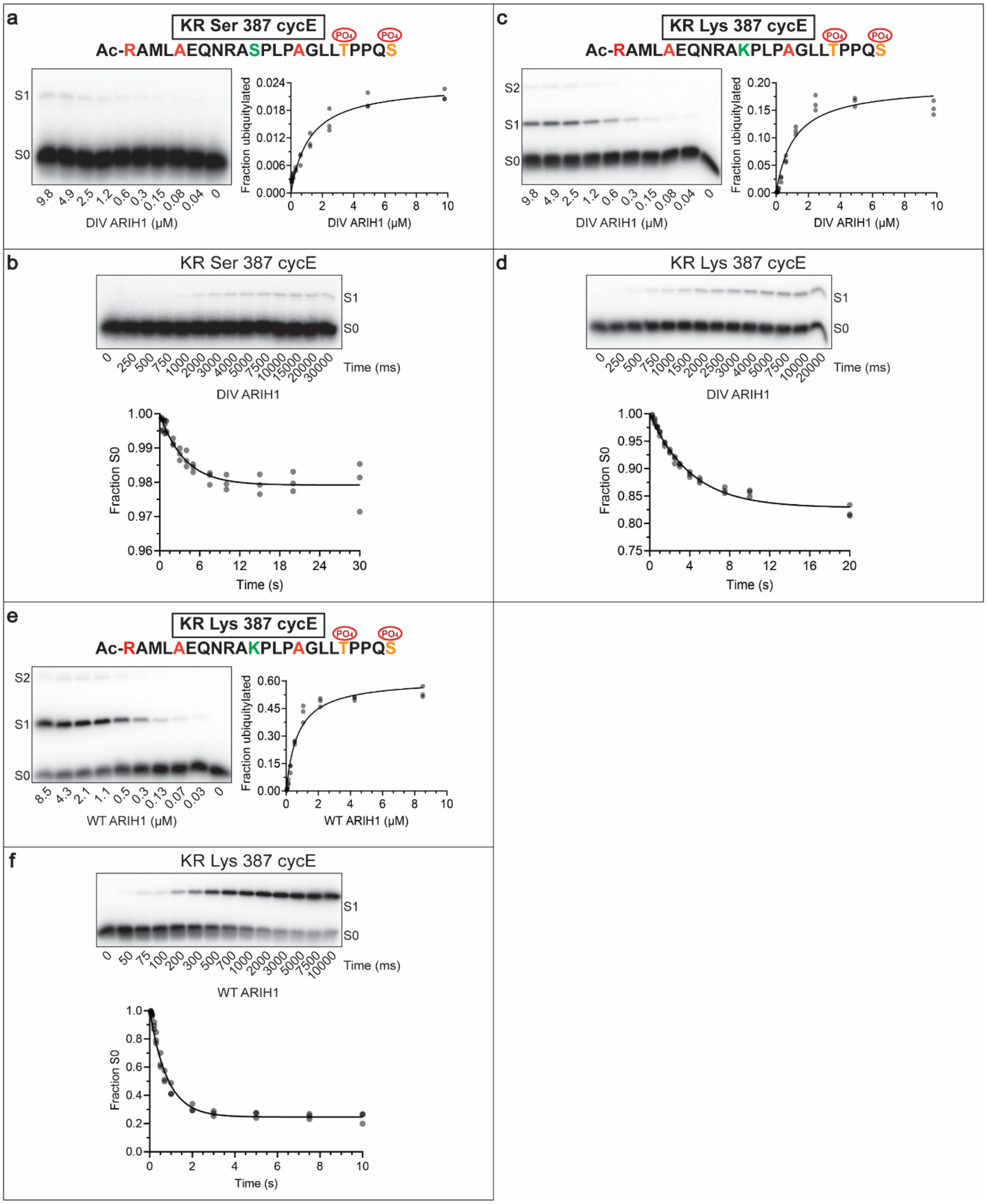

Supplementary Figure S9. Estimation of the kinetic parameters K_m and k_{obs} for WT or A397D/K398I/A399V 'DIV' ARIH1 with various cycE peptide-based ubiquitin acceptors. (a) Autoradiogram showing substrate (S0) conversion to product for ubiquitylation reactions containing increasing levels of DIV ARIH1 (left) and the fit of the data to the Michaelis-Menten equation (right) with the KR Ser 387 cycE peptide. (b) Autoradiogram showing a time course for

substrate (S0) conversion to product for pre-steady state ubiquitylation reactions (top) and the fit of the data to a closed form solution modeling the single-encounter reaction (bottom) with KR Ser 387 cycE peptide. (c) Same as (a) except with KR Lys 387 cycE. (d) Same as (b) except with KR Lys 387 cycE. (e) Same as (c) except with WT ARIH1. (f) Same as (d) except with WT ARIH1. All autoradiograms are representative of n=3 technical replicates.

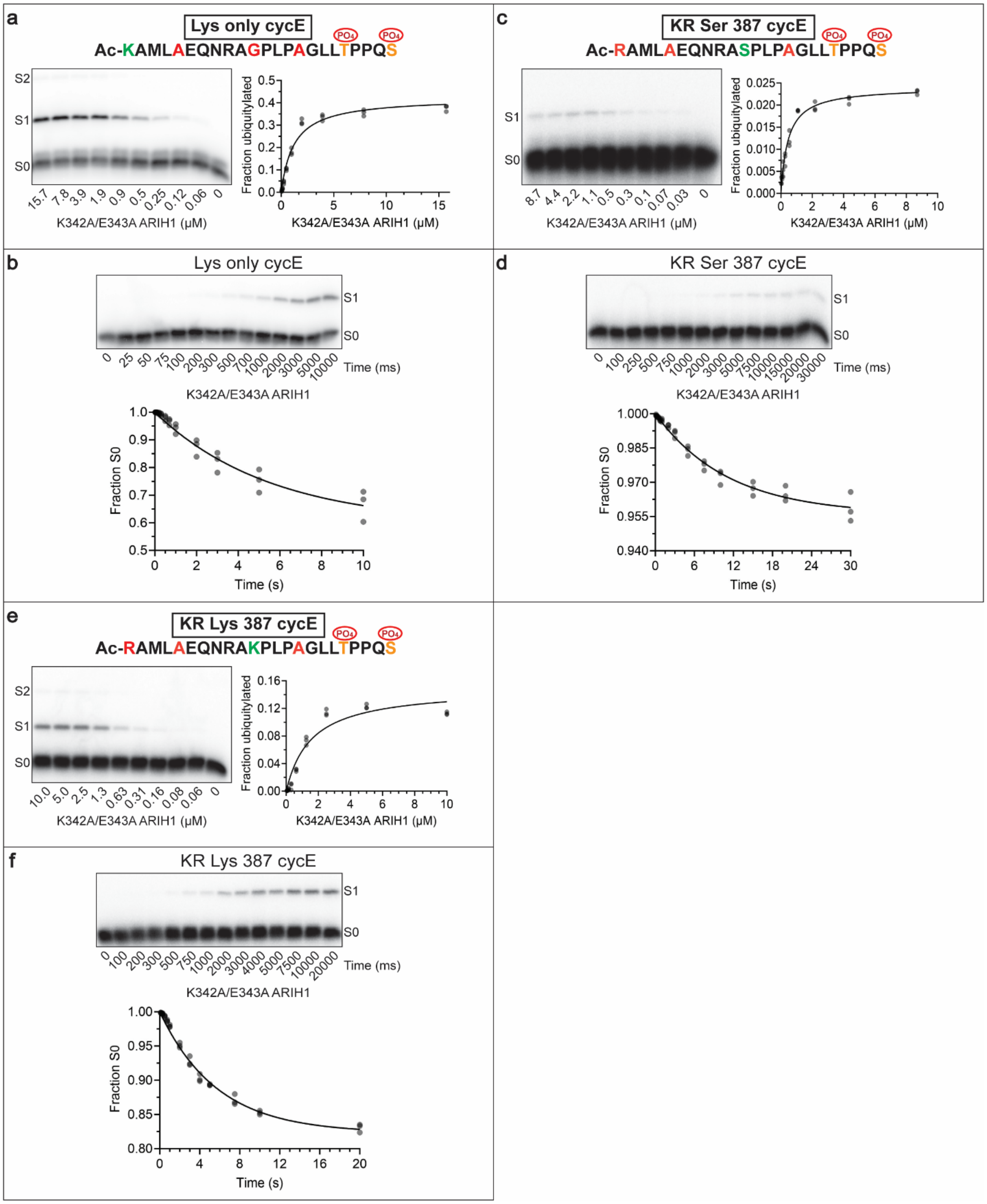

Supplementary Figure S10. Estimation of the kinetic parameters K_m and k_{obs} for K342A/E343A ARIH1 with various cycE peptide-based ubiquitin acceptors. (a) Autoradiogram showing substrate (S0) conversion to product for ubiquitylation reactions containing increasing levels of K342A/E343A ARIH1 (left) and the fit of the data to the Michaelis-Menten equation (right) with Lys only cycE peptide. (b) Autoradiogram showing a time course for

substrate (S0) conversion to product for pre-steady state ubiquitylation reactions (top) and the fit of the data to a closed form solution modeling the single-encounter reaction (bottom) with Lys only cycE peptide. (c) Same as (a) except with KR Ser 387 cycE. (d) Same as (b) except with KR Ser 387 cycE. (e) Same as (a) except with KR Lys 387 cycE peptide. (f) Same as (b) except with KR Lys 387 cycE peptide. All autoradiograms are representative of n=3 technical replicates.

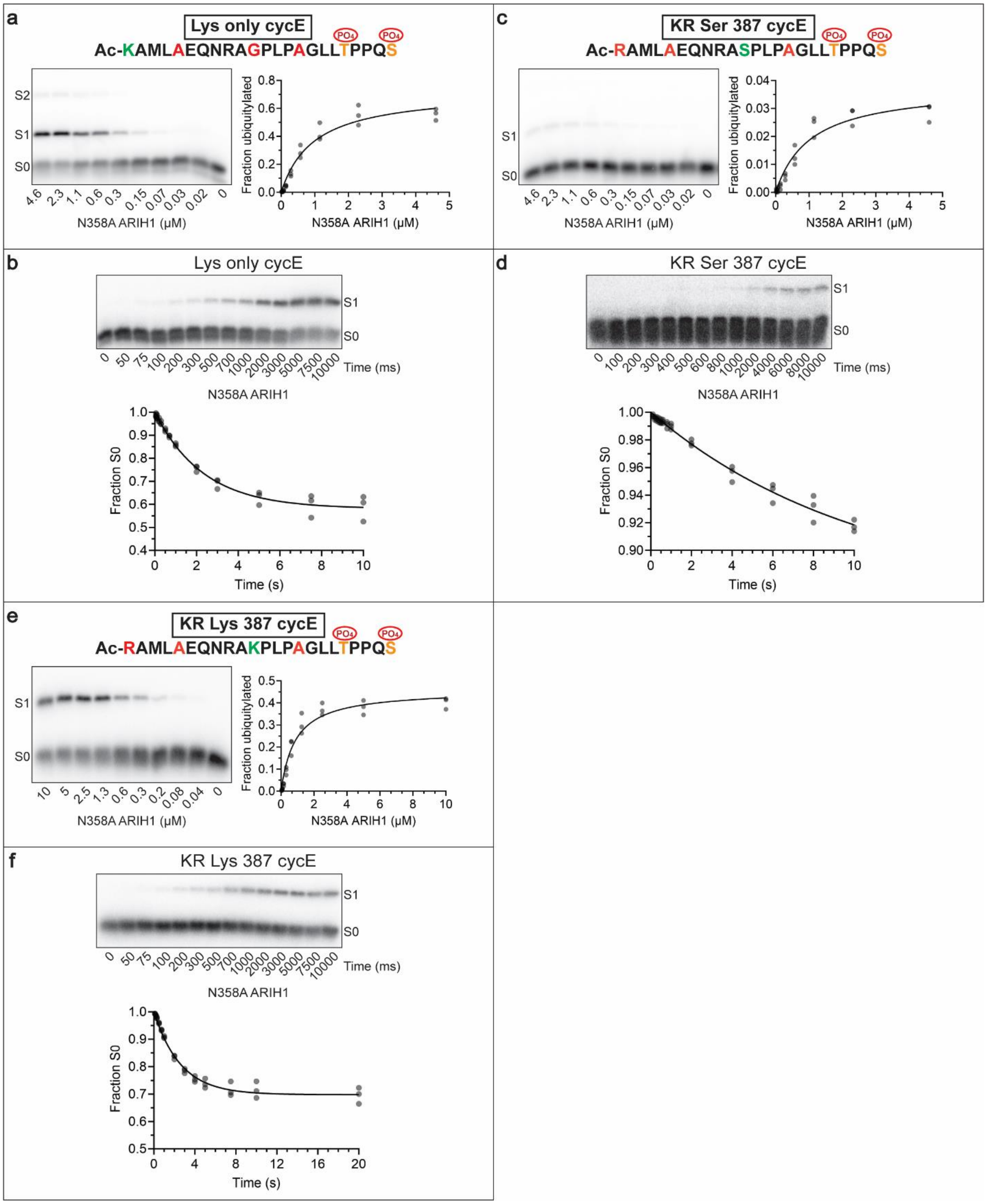

Supplementary Figure S11. Estimation of the kinetic parameters K_m and k_{obs} for N358A ARIH1 with various cycE peptide-based ubiquitin acceptors. (a) Autoradiogram showing substrate (S0) conversion to product for ubiquitylation reactions containing increasing levels of N358A ARIH1 (left) and the fit of the data to the Michaelis-Menten equation (right) with Lys only cycE peptide. (b) Autoradiogram showing a time course for substrate (S0) conversion to product

for pre-steady state ubiquitylation reactions (top) and the fit of the data to a closed form solution modeling the single-encounter reaction (bottom) with Lys only cycE peptide. (c) Same as (a) except with KR Ser 387 cycE. (d) Same as (b) except with KR Ser 387 cycE. (e) Same as (a) except with KR Lys 387 cycE peptide. (f) Same as (b) except with KR Lys 387 cycE peptide. All autoradiograms are representative of n=3 technical replicates.

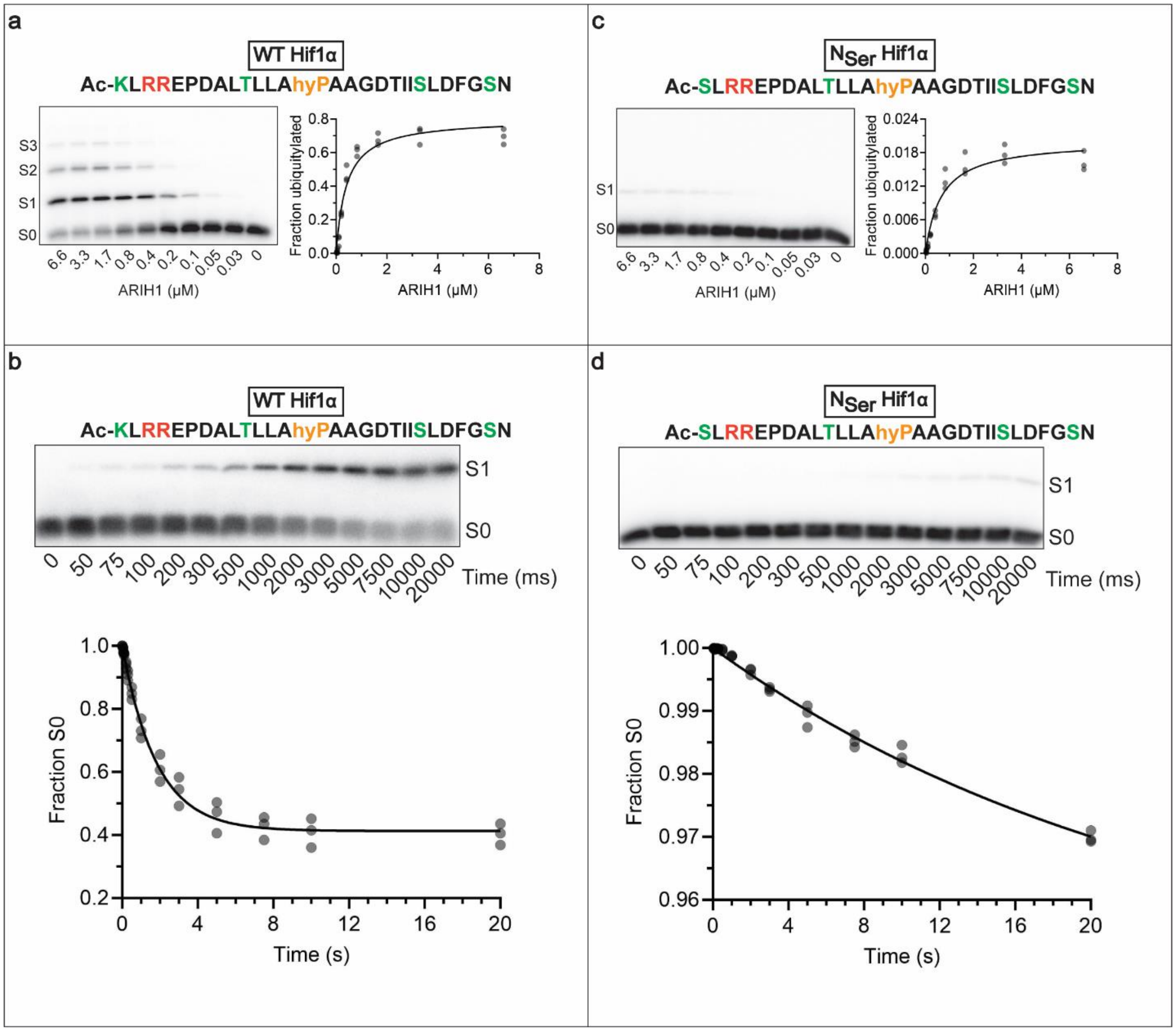

Supplementary Figure S12. Estimation of the kinetic parameters K_m and k_{obs} for CRL2^{VHL} and Hif1α peptide substrates. (a) Autoradiogram showing WT Hif1α substrate (S0) conversion to product for ubiquitylation reactions containing increasing levels of WT ARIH1 (left) and the fit of the data to the Michaelis-Menten equation (right). (b) Autoradiogram showing a time course for WT Hif1α substrate (S0) conversion to product for pre-steady state ubiquitylation reactions (top) and the fit of the data to a closed form solution modeling the single-encounter reaction (bottom) with WT ARIH1. (c) Same as (a) except with N_{ser} Hif1α substrate. (d) Same as (b) except with N_{ser} Hif1α substrate. All autoradiograms are representative of n=3 technical replicates.

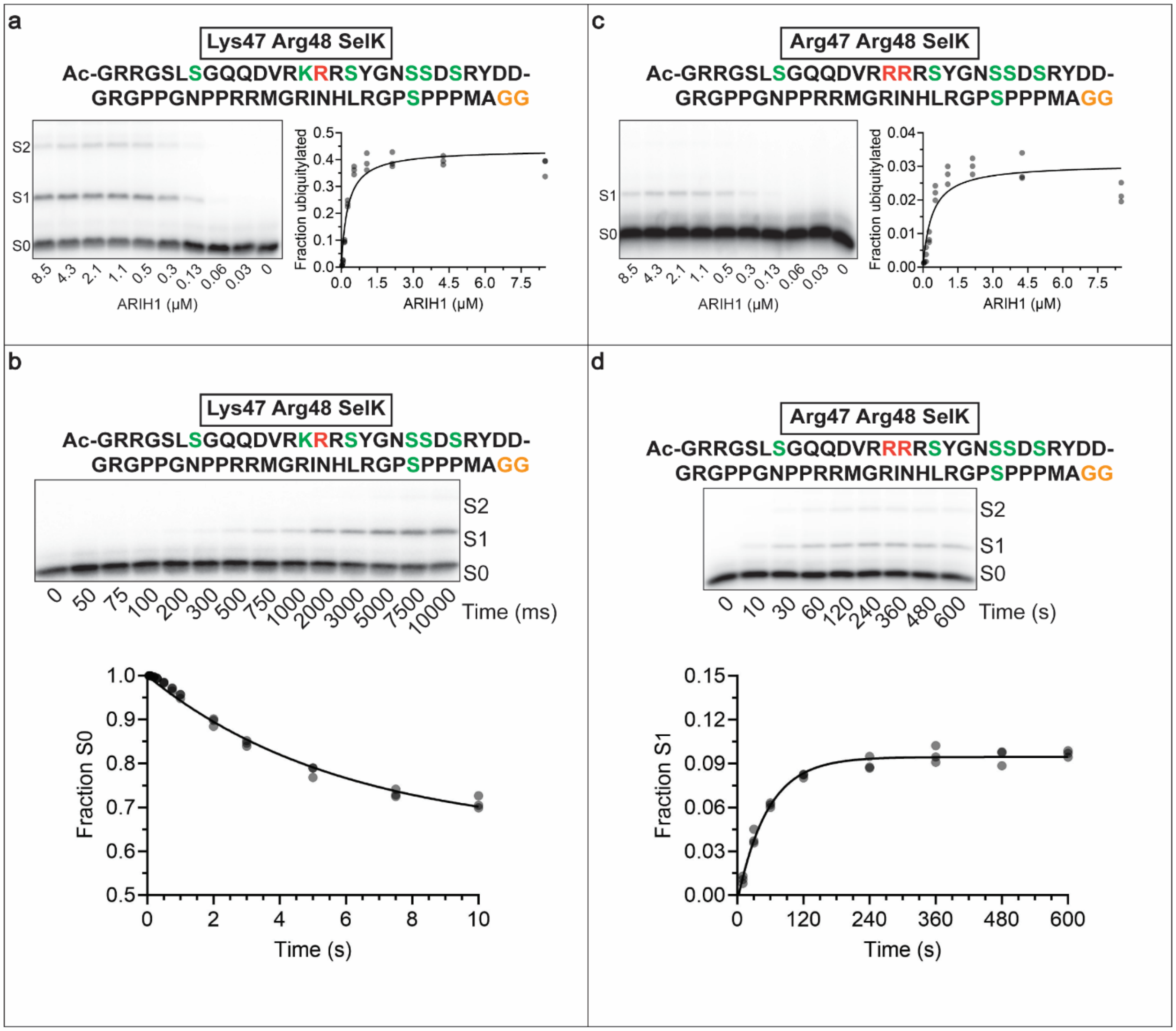

Supplementary Figure S13. Estimation of the kinetic parameters K_m and k_{obs} for CRL2^{KLHDC2} and SelK peptide substrates. (a) Autoradiogram showing Lys47 Arg48 SelK substrate (S0) conversion to product for ubiquitylation reactions containing increasing levels of WT ARIH1 (left) and the fit of the data to the Michaelis-Menten equation (right). (b) Autoradiogram showing a time course for Lys47 Arg48 SelK substrate (S0) conversion to product for pre-steady state ubiquitylation reactions (top) and the fit of the data to a closed form solution modeling the single-encounter reaction (bottom) with WT ARIH1. (c) Same as (a) except with Arg47 Arg48 SelK peptide. (d) Same as (b) except with Arg47 Arg48 SelK substrate. All autoradiograms are representative of n=3 technical replicates.
